# Abnormal ERPs and Brain Dynamics Mediate Basic Self Disturbance in Schizophrenia: A Review of EEG and MEG Studies

**DOI:** 10.3389/fpsyt.2021.642469

**Published:** 2021-04-12

**Authors:** Arthur Hamilton, Georg Northoff

**Affiliations:** ^1^Department of Cognitive Science, Carleton University, Ottawa, ON, Canada; ^2^Mind, Brain Imaging and Neuroethics Unit, Institute of Mental Health Research, University of Ottawa, Ottawa, ON, Canada

**Keywords:** schizophrenia, sense of self, basic self, self disturbance, electroencephalography, event-related potentials, magnetoencepalography

## Abstract

**Background:** Interest in disordered sense of self in schizophrenia has recently re-emerged in the literature. It has been proposed that there is a basic self disturbance, underlying the diagnostic symptoms of schizophrenia, in which the person's sense of being a bounded individual continuous through time loses stability. This disturbance has been documented phenomenologically and at the level of cognitive tasks. However, the neural correlates of basic self disorder in schizophrenia are poorly understood.

**Methods:** A search of PubMed was used to identify studies on self and schizophrenia that reported EEG or MEG data.

**Results:** Thirty-three studies were identified, 32 using EEG and one using MEG. Their operationalizations of the self were divided into six paradigms: self-monitoring for errors, proprioception, self-other integration, self-referential processing, aberrant salience, and source monitoring. Participants with schizophrenia were less accurate on self-referential processing tasks and had slower response times across most studies. Event-related potential amplitudes differed across many early and late components, with reduced N100 suppression in source monitoring paradigms being the most replicated finding. Several studies found differences in one or more frequency band, but no coherent overall finding emerged in this area. Various other measures of brain dynamics also showed differences in single studies. Only some of the study designs were adequate to establish a causal relationship between the self and EEG or MEG measures.

**Conclusion:** The broad range of changes suggests a global self disturbance at the neuronal level, possibly carried over from the resting state. Further studies that successfully isolate self-related effects are warranted to better understand the temporal-dynamic and spatial-topographic basis of self disorder and its relationship to basic self disturbance on the phenomenological level.

## Introduction

A range of approaches to the study of schizophrenia within psychiatry and psychology have converged on the concept of disordered experience of the self, also called self disorder (1). While self disorder has several phenomenological dimensions in schizophrenia (2), a large body of research shows that the basic self is disturbed (3). Such a basic disturbance has been thoroughly documented at the phenomenological level (4, 5) and, at the psychological level, subjects with schizophrenia have altered responses on a range of self-related cognitive tasks (6). The neural correlates of self disturbance remain unclear, however.

Basic self disorder (or basic self disturbance) is reflected phenomenologically in the loss of the basic experience of being oneself that underlies all normal awareness (4). The disturbance manifests as both hyper-reflexivity, the experience of oneself in the same way as the external world, and reduced self-affectation, the sense of oneself as a vital source of action and awareness (4). Ipseity or the sense of “mineness” is integral to ordinary experience but becomes disordered in patients with a basic self disturbance (7). To measure basic self disorder, the Examination of Anomalous Self-Experience (EASE) scale is most commonly employed (8).

Self disorder at the phenomenological level may be responsible for the following changes at the psychological level, among others. Subjects with schizophrenia are impaired in their source monitoring ability; that is, they struggle to distinguish between internally- and externally-generated events, actions, and voices (9). In addition, healthy subjects show a bias toward stronger performance on self-referential processing tasks, for instance more reliably remembering information about themselves than others, but those with schizophrenia show no advantage (10). Other abnormalities that may relate to basic self-disorder include impaired self-other integration, altered proprioception and exteroception, a reduced tendency to ascribe additional salience to aberrant stimuli, and impaired self-monitoring for errors (6, 11–13).

At the neuronal level, subjects with schizophrenia display several abnormalities measurable through electroencephalography (EEG), whether or not the task they are performing relates to the self. These include a reduced difference in amplitudes between an initial stimulus and a second, repeated stimulus in both P50 and N100 event-related potential (ERP) components, before and after symptoms emerge (14). Subjects with schizophrenia also exhibit reduced mismatched negativity (MMN), P300, and P3a amplitude (14). Resting-state studies on subjects with schizophrenia have shown increased power in the delta and theta frequency bands and decreased power in the alpha frequency band (15).

The goal of this article is to review the existing EEG and MEG literature on schizophrenia and the self, in order to identify the magnetoelectrical, and possibly dynamic, basis of basic self-disorder in schizophrenia. Firstly, the range of experimental paradigms employed is surveyed, and the behavioral measures from those paradigms are reviewed. Secondly, ERP component results from the different paradigms are presented. Thirdly, dynamic measures including frequency bands and others are surveyed. Implications of the findings and limitations of the present review are then discussed.

## Methods

Due to highly heterogeneous study designs in the literature, the article takes the form of a narrative review. It is based on a survey of the database PubMed in October 2020. “Self” and “schizophrenia” were used as search terms in combination with “electroencephalography” and “magnetoencephalography.” Articles were selected for inclusion if they met three criteria: (1) appropriate participants, (2) appropriate methods, and (3) appropriate topic.

Regarding participants, studies were included if subjects with schizophrenia were included. Studies of patients with other forms of psychosis or related personality disorders were excluded to increase the degree of homogeneity among the studies, though studies covering multiple types of psychopathology including schizophrenia were included. Studies on schizotypy in healthy individuals were excluded.

Regarding methods, studies using EEG were included, as well as studies using the related method of magnetoencephalography (MEG). Some studies used other neuroimaging methods such as functional magnetic resonance imaging (fMRI) and diffusion tensor imaging (DTI) in addition to EEG or MEG, but results from these other modalities are not covered in this review.

Regarding topic, articles in which authors described themselves as studying the self were included. This led to a broad conceptualization of the self and the possible inclusion of studies that did not in fact measure self-related effects. However, removing studies whose authors deemed them self-related would have risked eliminating genuine self-related findings from the review; the current approach was thus deemed preferable.

## Results

The search yielded 33 relevant studies. One study used MEG (16), while the remaining 32 used EEG. Greater detail on the participants, study designs, and results of all the studies can be found in the Supplementary Material. The studies used a range of different tasks and many reported comparative data on accuracy and response times. These studies have also investigated a range of neuronal measures, including ERP component amplitudes and latencies, several measures from different frequency bands, and various other dynamic measures, including prestimulus and during a resting state.

### Paradigms or Tasks Probing the Self and Behavioral Data

**Figure 1:**
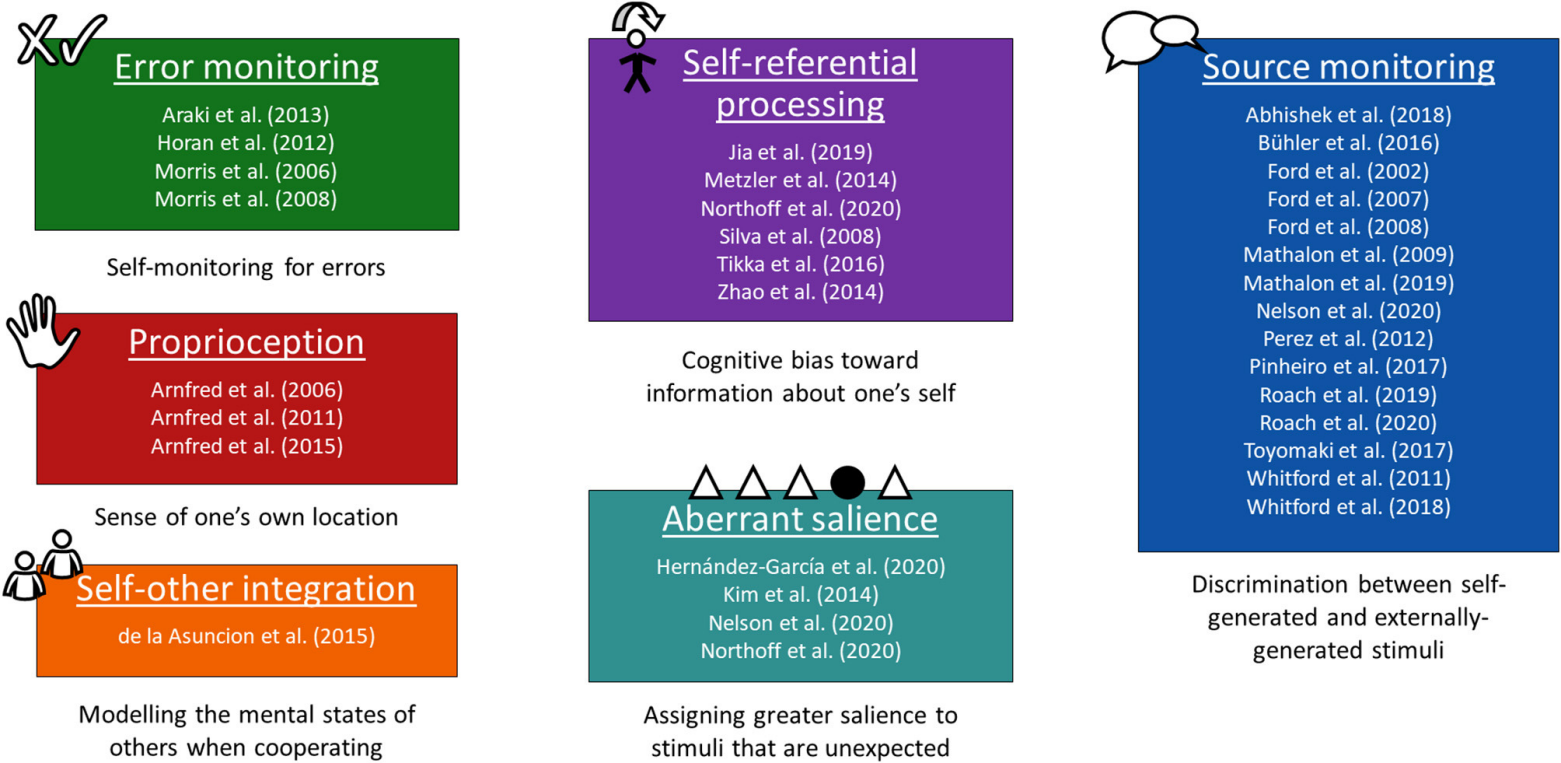
Studies divided by experimental paradigm. The studies that were selected for inclusion in the literature review grouped into six paradigms based on the type of self-related task used in their experimental design.

The studies obtained their data using a range of different tasks that their authors deemed self-related. Similar tasks are grouped together into six over-arching paradigms in Figure 1 (16–46) based on the aspect of self involved. It must be borne in mind, however, that these tasks may also draw to varying extents on mental faculties other than sense of self. Two studies used tasks from more than one of these paradigms (27, 32). Error-monitoring was measured using tasks such as the Stroop task (17). The studies on proprioception applied small amounts of weight to participants' hands (21–23). Self-other integration was assessed with a social Simon task, a variant of the Simon task featuring an individual condition and a joint condition in which some stimuli are for one participant and some are for the other (24). For self-referential processing, studies used various tasks, for instance asking participants to judge whether adjectives (1) describe them, or (2) describe a person they know, and testing which group of adjectives they later remember better (25, 26, 30). Sensitivity to aberrant salience was measured through tasks such as an auditory oddball task (27, 31). Source monitoring accounted for the most articles, which were predominantly from Ford and colleagues, who used a simple task with (1) a talk condition, and (2) a listen condition in which the recording of the talk condition is played back to the participant (36–40, 42, 43, 45, 46). In addition to these paradigms, some studies presented resting-state data and/or data on the difference between the resting state and task-related activity (16, 27, 29, 47, 48).

**Figure 2:**
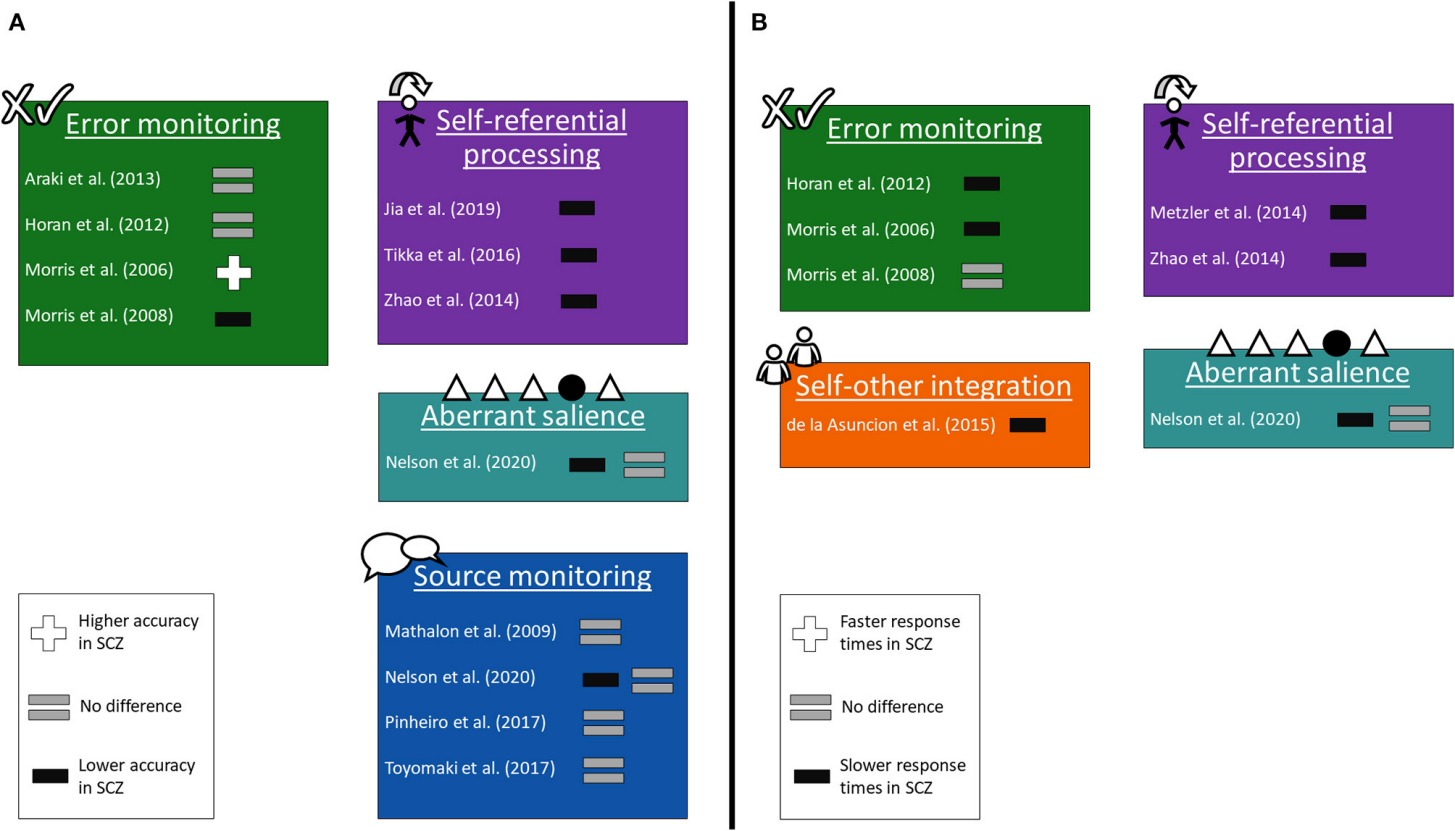
Changes in behavioral responses. The studies grouped into the same paradigms as in Figure 1, showing the differences between subjects with schizophrenia and healthy controls in task responses. **(A)** Differences in accuracy. **(B)** Differences in response times.

Many studies provided behavioral data from their respective paradigms (17–20, 24–26, 29, 30, 32, 39, 41, 44), which is presented in Figure 2.

### Event-Related Potentials Data

**Figure 3:**
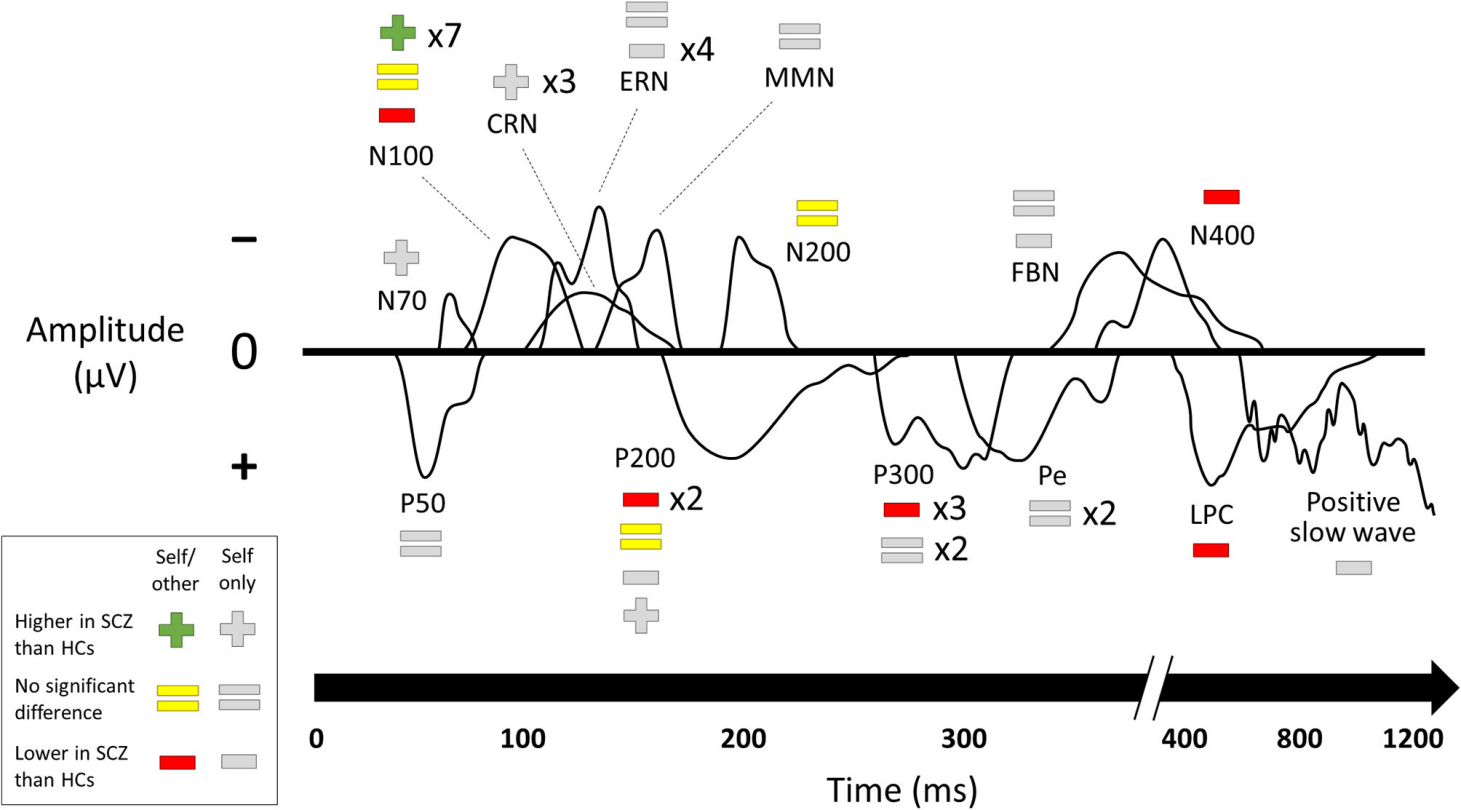
Changes in ERP component amplitudes including null results. Studies that compared ERP component amplitudes in subjects with schizophrenia to those in healthy controls. SCZ, subjects with schizophrenia. HCs, healthy controls. Multipliers beside a plus, equals, or minus sign indicate the number of studies that reported this result. Studies marked as “self/other” compared self-related task data to comparable non-self-related task data to establish a role for the self. Studies marked as “self only” used a self-related task but without a control condition to establish that it was this aspect of the task that caused the ERP component change. The visual depictions of ERP components are stylized approximations that do not correspond to the findings of any of the studies in particular. A few ERP components were not named in the studies and were categorized here based on their latency and whether they were positive- or negative-going. Results of the individual studies can be found in the Supplementary Material.

**Figure 4:**
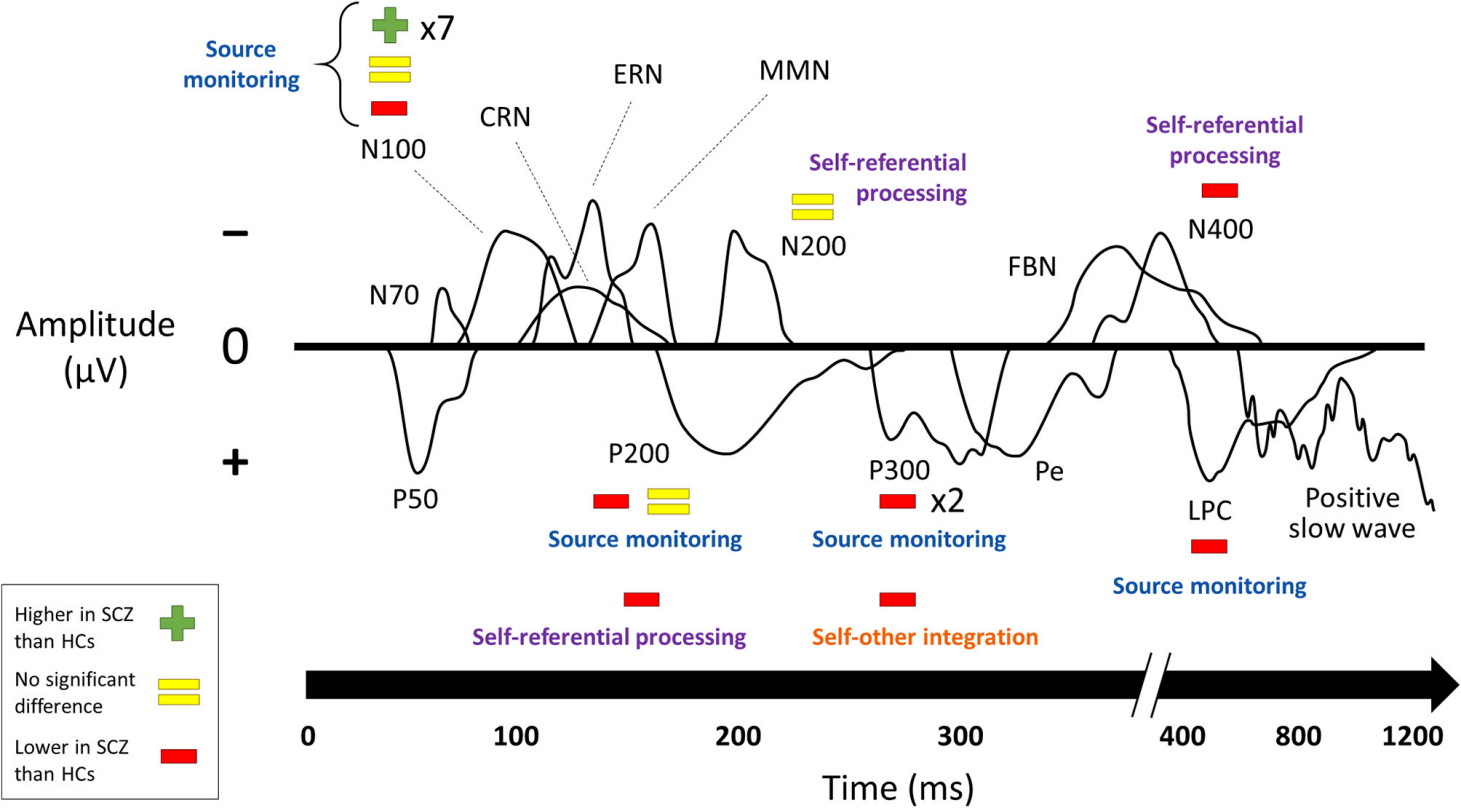
Changes in ERP component amplitudes by paradigm. Studies that compared ERP component amplitudes in schizophrenia to those in healthy controls, excluding those that did not compare self-related task data to comparable non-self-related task data. Sorted according to the paradigms from Figure 1. SCZ, subjects with schizophrenia. HCs, healthy controls. Multipliers beside a plus, equals, or minus sign indicate the number of studies that reported this result. The visual depictions of ERP components are stylized approximations that do not correspond to the findings of any of the studies in particular. A few ERP components were not named in the studies and were categorized here based on their latency and whether they were positive- or negative-going. Results of the individual studies can be found in the Supplementary Material.

Results of comparisons between patients with schizophrenia and healthy controls in ERP component amplitudes (17–21, 24, 26, 28, 30, 32–34, 36, 38–41, 43–46) are summarized in Figure 3. Results from the subset of comparisons that compared self-related task data to similar non-self-related task data are connected to the behavioral paradigms from above in Figure 4.

A few studies attempted to find correlations between ERP component amplitudes and scores from psychopathology scales within their group of patients with schizophrenia, yielding a mixture of significant correlations (positive and negative) and null results. Metzler et al. (26) found that as psychopathology scores (measured through a relevant subset of items from the Scale for the Assessment of Positive Symptoms scale) increased, the difference between conditions in N400 amplitude decreased. Nelson et al. (32) found several significant findings linking neurocognitive and psychopathological measures, most notably that source monitoring results (using a composite of psychological and ERP measures) accounted for 39.8% of the variance in EASE scores. In certain experimental conditions, Pinheiro et al. (41) found two modestly significant correlations between late positive component (LPC) amplitude and psychopathology scales relating to hallucinations after correcting for multiple comparisons. Mathalon et al. (39) found that reduced N100 suppression was correlated with unusual thought content in one of two patient groups. Bühler et al. (34), by contrast, found no correlation between Positive and Negative Syndrome Scale (PANSS) scores and either N100 amplitude or the amplitude of an unidentified “late component” that appears to be a P200. Perez et al. (40) likewise found no correlation between N100 suppression and any of four psychopathology subscales.

Only four studies measured ERP component latencies in patients with schizophrenia and healthy controls. Arnfred et al. (21) used a proprioception task and found higher P60 latencies in subjects with schizophrenia than healthy controls. Using a self-referential processing paradigm, Zhao et al. (30) found significantly higher P200 latencies in subjects with schizophrenia but no difference in N200 latencies. Finally, on source monitoring tasks, Whitford et al. (45) found no difference in N100 latencies and Abhishek et al. (33) found no difference in P300 latencies. No studies attempted to correlate ERP component latencies with scores on psychopathology scales.

### Measures of Brain Dynamics

**Figure 5:**
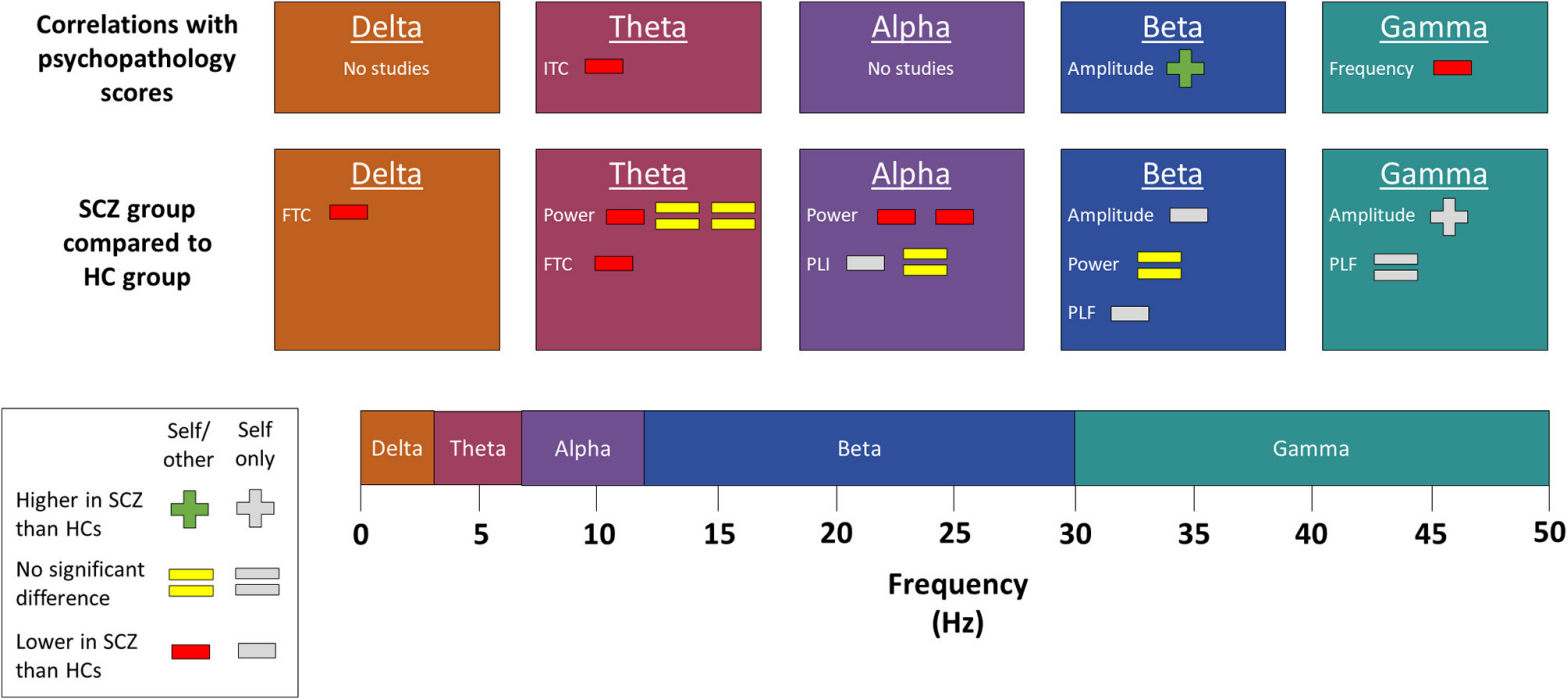
Changes in individual frequency bands. Results comparing individual frequency bands in subjects with schizophrenia to those in healthy controls. SCZ, subjects with schizophrenia. HCs, healthy controls. FTC, frontotemporal coherence. ITC, intertrial coherence. PLI, phase lag index. PLF, phase-locking factor. The presence of multiple signs beside one measure indicates multiple separate results. Studies marked as “self/other” either compared self-related task data to similar non-self-related task data or else correlated their findings with a psychopathology scale that measures self disorder. By contrast, studies marked as “self only” used neither of those methods to establish whether the observed change was related to the self. Most of the studies included in this figure used a source monitoring paradigm. However, the following results were from other paradigms. Self-referential processing paradigms were used by Jia et al. (25), who found no difference in theta or beta power but decreased alpha power and phase lag index. Proprioception paradigms were used by Arnfred et al. (22), who found decreased beta amplitude and phase-locking factor, increased gamma amplitude, and no difference in gamma phase-locking factor, and Arnfred et al. (23), who found a positive correlation between beta amplitude and psychopathology scales but a negative correlation between gamma frequency and psychopathology scales.

**Figure 6:**
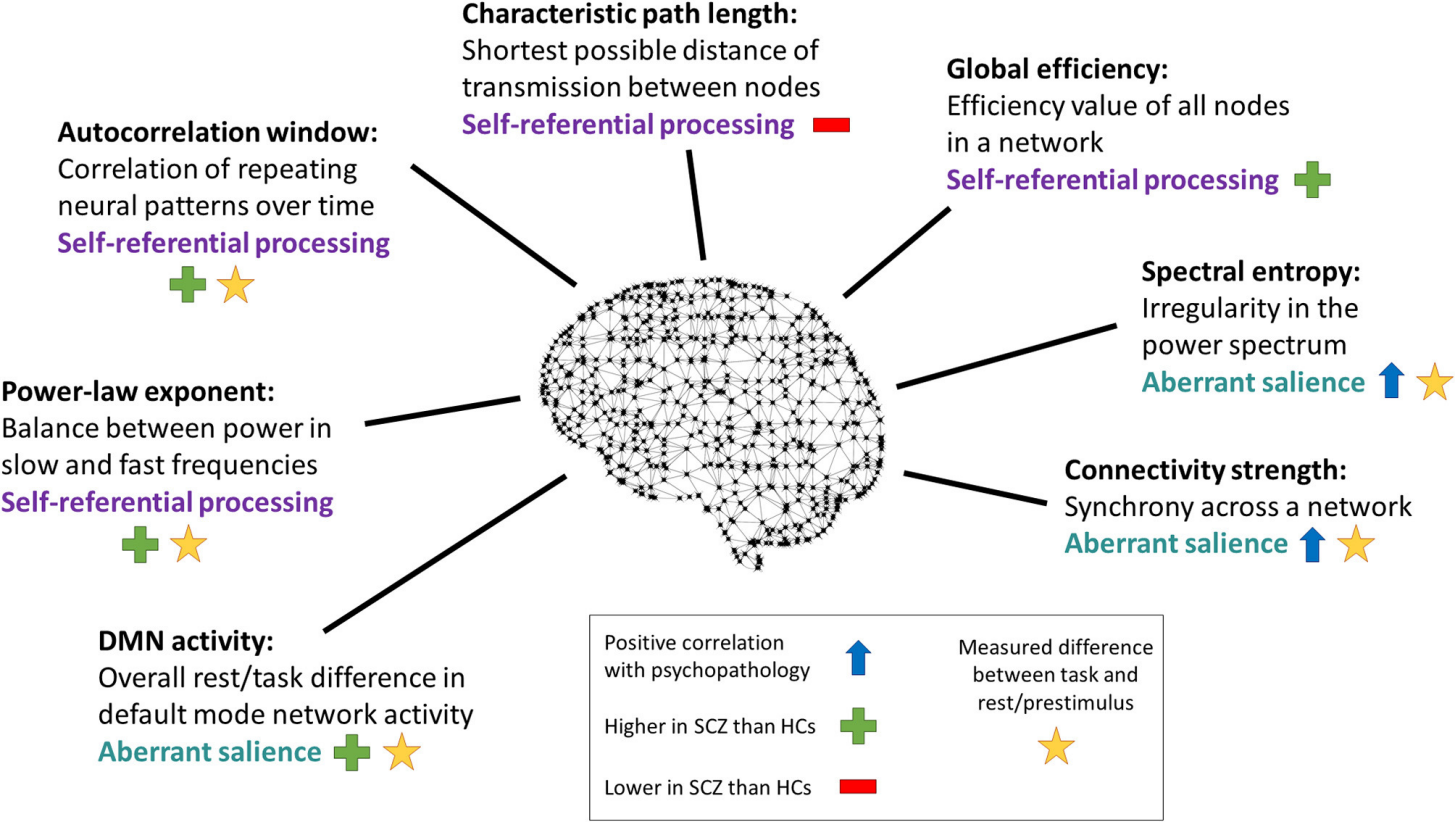
Changes in other dynamic measures. Dynamic measures studied that were not individual frequency bands, labeled with the paradigm used to obtain them. SCZ, subjects with schizophrenia. HCs, healthy controls. With the exception of the study by Kim et al. (16), all of these studies differentiated self-related effects from non-self-related effects either through comparison of experimental conditions or through correlational analyses with psychopathology scales that measure self disorder.

The findings of studies investigating one or more individual frequency bands (22, 23, 25, 35, 43, 44) are summarized in Figure 5 (excluding results from prestimulus or a resting state). In addition, Kim et al. (16) found increased theta, alpha, and beta rest-task difference in subjects with schizophrenia in the posterior cingulate cortex, as well as decreased gamma rest-task difference in the medial prefrontal cortex. A number of other dynamic EEG measures have also been applied to self and schizophrenia (16, 25, 27, 31), and these are summarized in Figure 6. Northoff et al. (27) also performed a moderation analysis and found that when autocorrelation window (ACW) and power-law exponent (PLE) values were low, self disorder (measured phenomenologically) was inversely related to negative schizophrenia symptoms, whereas when ACW and PLE were high, self disorder was positively correlated with schizophrenia.

**Figure 7:**
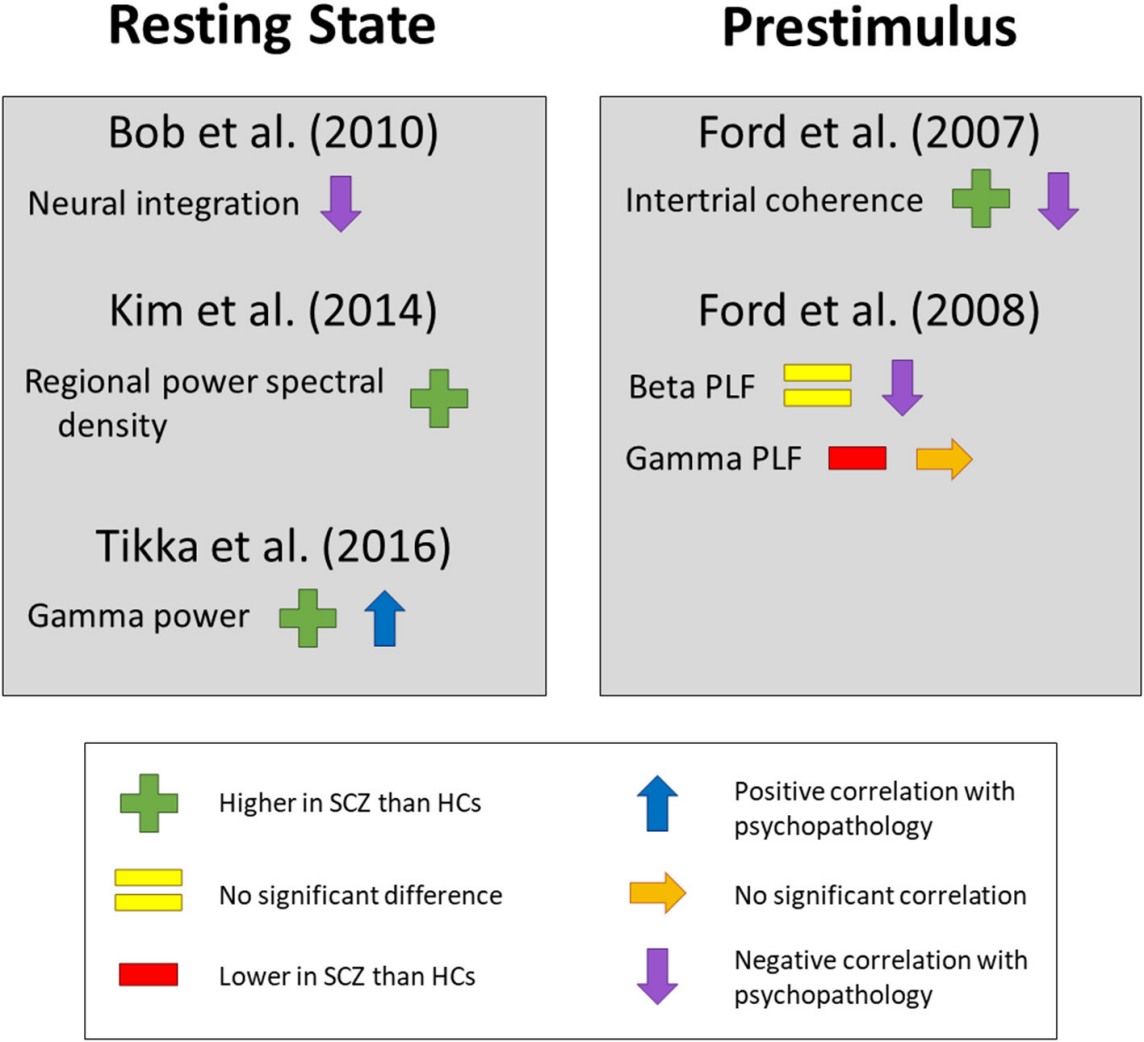
Changes in resting state and prestimulus measures. Findings on the resting state and prestimulus period, including comparisons of subjects with schizophrenia to healthy controls and correlations with psychopathology scales. SCZ, subjects with schizophrenia. HCs, healthy controls. PLF, phase-locking factor. Neural integration (47) was computed as correlations between 16 electrode pairs. For the resting state findings, a direct link to the self was established only by Bob et al. (47), by correlation with the Dissociative Experiences Scale. For the prestimulus findings, a direct link to the self was established only by Ford et al. (2007) in their comparison of patients and controls, through an interaction effect with experimental condition. This figure does not show findings from studies examining rest/task differences or prestimulus/task differences, as these were shown in Figure 6.

Figure 7 summarizes results that were obtained either prestimulus or during a resting state (16, 29, 36, 37, 47), including measures from both individual frequency bands and other dynamic measures. In addition, Kindler et al. (48) compared periods with auditory verbal hallucinations to periods when these were not present in a resting-state study of EEG microstates and found that a specific class of microstates situated in the frontocentral region was significantly shorter during auditory verbal hallucinations.

## Discussion

This article has reviewed the literature on EEG and MEG, schizophrenia, and self. A range of studies have showed alterations across a range of ERP components in multiple paradigms, differences in delta, theta, alpha, beta, and gamma frequency bands, changes in seven other dynamic measures, and differences in resting state and prestimulus measures. Some of these results, in particular many from ERP studies and resting state or prestimulus studies, despite using self-related paradigms, have not directly established that the self-related aspect of the paradigm is responsible for the differences, i.e., through a comparable non-self-related condition or correlation with psychopathology scales measuring self disorder. Nonetheless, the role of the self was established in enough studies to show significant differences in both early and late ERP components, across all frequency bands, and in six other dynamic measures. These findings show that disordered sense of self in patients with schizophrenia is correlated with a diverse set of event-related and dynamic neuronal measures. The diversity of these measures in turn suggests that, at the neuronal level, this disorder of self is global in nature and always present, which aligns with the phenomenological finding of basic self disorder.

### Behavior and Event-Related Potentials

At the behavioral level, the findings reveal differences between patients with schizophrenia and healthy controls, though not universally. Patients scored lower in terms of accuracy on self-referential processing tasks, but not on most source monitoring tasks or other paradigms. This result suggests performance on self-related tasks in subjects with schizophrenia is impaired in some areas but not others. Conversely, response times were mostly slower in subjects with schizophrenia than in healthy controls, across the four paradigms for which data was available. This finding suggests a slower rate of processing on self-related tasks in schizophrenia. However, it is difficult to dissociate findings on processing-speed efficiency in schizophrenia from the effects of antipsychotic medication, higher doses of which correlate with lower processing speed (49).

Studies of ERPs reveal changes in schizophrenia across multiple paradigms and both early and late components. Many of these compared self-related and non-self-related experimental conditions in order to specifically establish a relationship with the self. These studies were mainly from two paradigms: source monitoring and self-referential processing. By far the best-established change in an ERP component is the reduction of N100 suppression (that is, the reduction of the difference in N100 amplitude between speaking and listening conditions) in subjects with schizophrenia on source-monitoring tasks. The other finding for which the role of self was directly established in the source monitoring paradigm was reduced amplitude in schizophrenia in later components (P200, P300, and LPC). Studies on self-referential processing tasks for which the role of self was directly established also found lower amplitudes on later components (P200 and N400). The focus on later components for the self-referential processing paradigm likely reflects the higher-level cognitive processing involved in such tasks.

### Neuronal Dynamics

Several studies that investigated brain dynamics in one or more frequency bands found significant differences between subjects with schizophrenia and healthy controls during self-related paradigms. There is no clear trend across studies, especially once studies that did not directly establish a link to the self are excluded. Power and coherence may be decreased in schizophrenia in lower frequencies. However, the small number of studies available, the lack of a consistent measure of coherence, and exceptions to the trend for power prevent any specific conclusions from being drawn. Nonetheless, many studies did find significant differences between subjects with schizophrenia and healthy controls, indicating that self disorder has a dynamic basis.

Changes in various other measures of brain dynamics have been observed using self-related paradigms in schizophrenia, although only in single studies. Differences in these more holistic measures align with the finding that changes are found across all frequency bands and suggest a more global dynamic disturbance underlying self-disorder in schizophrenia. Future research should further investigate these and other dynamic measures, with the goal of identifying the changes underlying basic self disturbance in the dynamic neural pattern.

Studies from prestimulus or a resting state have found a range of changes in schizophrenia. Most of these, however, did not compare to a non-self-related condition or use a psychopathology scale that measures self disorder specifically, and no studies have yet sought to replicate any of these findings. Conclusions about the specific relationship between basic self disorder and dynamic non-task-related activity in schizophrenia cannot therefore be drawn at this time. Nonetheless, almost all studies did find changes in the resting state or prestimulus period, including the two that tested the role of the self directly. This suggests that changes to the self in schizophrenia have a neural basis that precedes any specific task.

### Implications

Overall, the range of EEG and MEG measures on which patients with schizophrenia differ from healthy controls and the range of self-related paradigms across which these differences emerge suggest that self-disorder in schizophrenia is not limited to a particular cognitive domain. Instead, the underlying sense of self appears to be domain general. This overall finding remains even when studies that claimed a connection to the self but did not directly establish it are excluded.

One theory that accounts for this global nature of the self is the basic model of the self, which postulates that sense of self arises from the spontaneous activity of the brain as a basic feature rather than as a cognitive representation (50). This theory aligns with Spatiotemporal Neuroscience, which proposes that underlying the brain's more readily observable ability to engage in cognition is a deeper layer of spatiotemporal dynamics present both at rest and during task-related processes (51, 52). The basic self would be situated at this level and hence schizophrenia would be fundamentally a disorder of the brain's underlying spatiotemporal dynamics rather than any particular cognitive domain (53).

**Figure 8:**
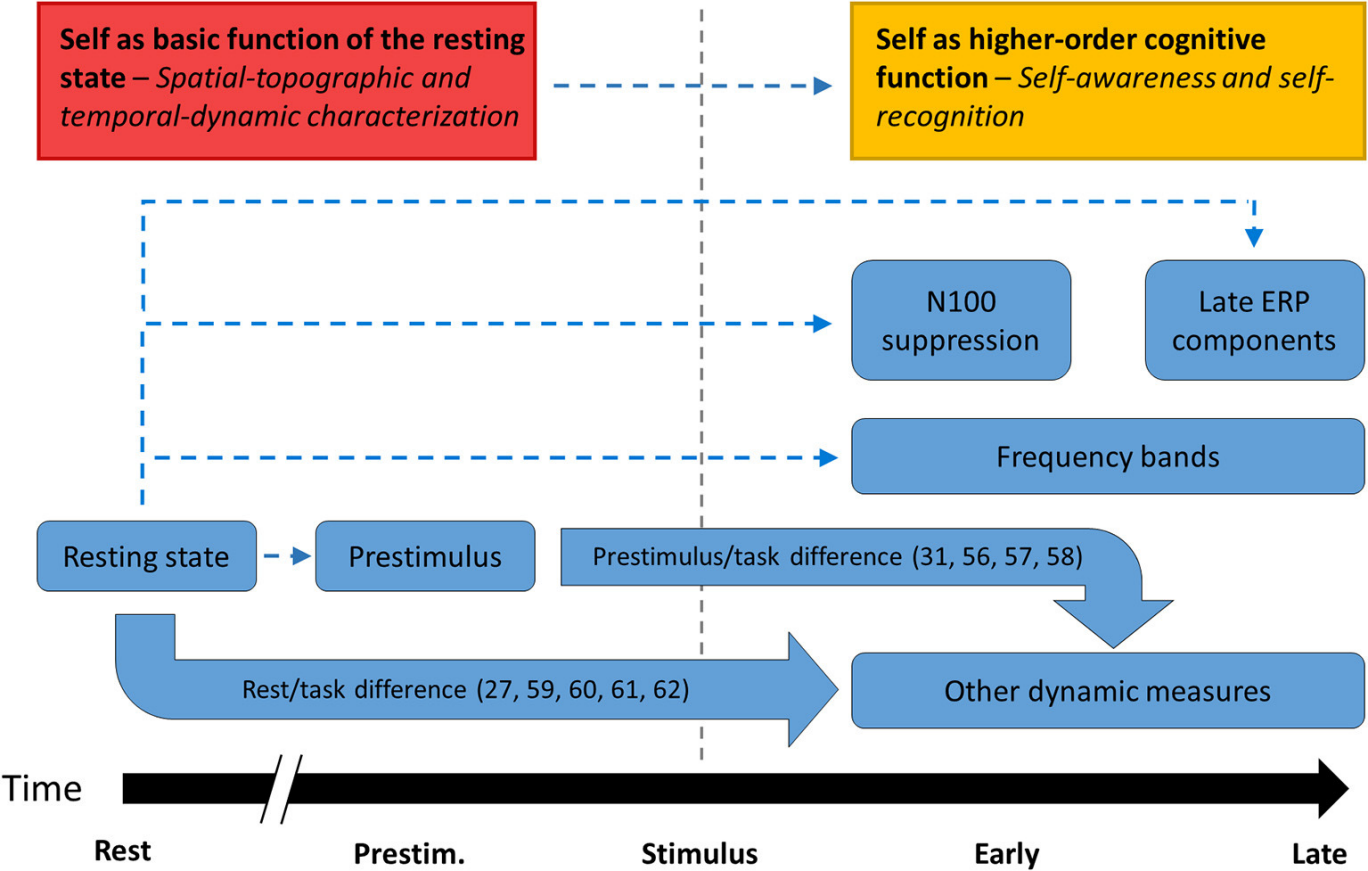
Proposed relationships between empirical findings. The major groups of findings identified in this review. Thick, solid arrows indicate relationships that were studied empirically in the literature while thin, dashed arrows indicate relationships that the basic model of self proposes in order to unify separate findings in the literature.

More specifically, we suggest that the neuronal distinction between resting state/prestimulus activity and task-related activity underlies what is described as a self/other distinction at the cognitive level. Changes to the rest/task difference on neuronal measures could thus manifest as a failure to demarcate self and other, or self disorder. For instance, the typical human ability to engage in self-referential processing more reliably than comparable non-self-referential processing may depend on a typical rest-task difference in the brain, which is then impaired in schizophrenia (25, 29, 30). The basic model of self thus incorporates the view that sense of self is a metacognitive faculty that is compromised in schizophrenia (54, 55) while also situating this faculty in relationship to the resting state activity of the brain. Figure 8 shows a conceptual schema of how the different types of neuronal findings covered by this review may be related, indicating connections directly supported by the findings [and related literature (56–62)] as well as proposed connections that would integrate the findings into a parsimonious theory.

### Methodological Limitations

The present review has certain limitations. Studies were selected for inclusion based on whether they conceptualized their study as relating to sense of self, but some other studies may have used similar paradigms without referring to sense of self and thus been excluded. Also, due to heterogeneous study designs and findings, main effects for participant group and interaction effects between participant group and self/other condition were sometimes grouped together in the figures with interaction effects with other variables such as electrode site. Nonetheless, the distinction in the figures between studies that established the distinction between self-related and non-self-related effects and those that did not meant the most important interaction effects were addressed. The study also did not generally attempt to localize brain processes relating to the self in subjects with schizophrenia because EEG does not provide fine-grained spatial resolution and there was only one MEG study. Another consequence of heterogeneous study methodologies was that meta-analytic statistical techniques could not be applied and replication was described simply in terms of the number of studies with similar findings.

Apart from reduced N100 suppression, all the neuronal changes observed require further replication, and the N100 suppression finding should be studied with alternate study designs to assess its validity outside talk-listen designs. Researchers studying self disorder in schizophrenia should remember to examine differences between self-related task data and comparable non-self-related task data in order to better isolate sense of self from other processes activated by the paradigms. Future electrophysiological research on self and schizophrenia should also investigate under-studied measures such as ERP component latencies, correlations between ERP amplitudes and psychopathology scales, and rest-task differences (63) in frequency band measures and other dynamic measures. Given the range of measures on which changes were observed, researchers should also search for other more comprehensive temporal-dynamic and spatial-topographic measures that could account for the profusion of existing findings more parsimoniously. These new neurodynamic measures of basic self disorder may ultimately be validated for use in the diagnosis and treatment of schizophrenia.

## Conclusion

There is good evidence that the self-disturbance seen in schizophrenia at phenomenological and psychological levels is mediated by changes at the neuronal level. While many details of the magnetoelectrical changes underlying self disorder remain tentative, the changes do appear to occur across a range of EEG and MEG measures in a more-or-less domain-general manner, that is, across different tasks and paradigms. This domain generality is suggestive of a global neuronal disturbance, which would account for the pervasive nature of the basic self disturbance on the phenomenological level.

## Author Contributions

AH was responsible for selecting studies for inclusion, writing the text, and creating the Figures and Supplementary Material. GN provided detailed feedback throughout the research process and written comments on article drafts. All authors contributed to the article and approved the submitted version.

## Conflict of Interest

The authors declare that the research was conducted in the absence of any commercial or financial relationships that could be construed as a potential conflict of interest.

## References

[1] Lysaker PH Lysaker JT. Schizophrenia and alterations in self-experience: a comparison of 6 perspectives. Schizophr Bull. (2010) 36:331–40. 10.1093/schbul/sbn077 18635676 PMC2833111

[2] Sass L Borda JP Madeira L Pienkos E Nelson B. Varieties of self disorder: a bio-pheno-social model of schizophrenia. Schizophr Bull. (2018) 44:720–7. 10.1093/schbul/sby001 29529266 PMC6007751

[3] Hur JW Kwon JS Lee TY Park S. The crisis of minimal self-awareness in schizophrenia: a meta-analytic review. Schizophr Res. (2014) 152:58–64. 10.1016/j.schres.2013.08.042 24055201

[4] Sass LA Parnas J. Schizophrenia, consciousness, and the self. Schizophr Bull. (2003) 29:427–44. 10.1093/oxfordjournals.schbul.a007017 14609238

[5] Nelson B Raballo A. Basic self-disturbance in the schizophrenia spectrum: taking stock and moving forward. Psychopathology. (2015) 48:301–9. 10.1159/000437211 26368118

[6] van der Weiden A Prikken M van Haren NEM. Self-other integration and distinction in schizophrenia: a theoretical analysis and a review of the evidence. Neurosci Biobehav Rev. (2015) 57:220–37. 10.1016/j.neubiorev.2015.09.004 26365106

[7] Nelson B Parnas J Sass LA. Disturbance of minimal self (ipseity) in schizophrenia: clarification and current status. Schizophr Bull. (2014) 40:479–82. 10.1093/schbul/sbu034 24619534 PMC3984529

[8] Parnas J Møller P Kircher T Thalbitzer J Jansson L Handest P. EASE: examination of anomalous self-experience. Psychopathology. (2005) 38:326–58. 10.1037/t30080-000 16179811

[9] Nelson B Whitford TJ Lavoie S Sass LA. What are the neurocognitive correlates of basic self-disturbance in schizophrenia?: integrating phenomenology and neurocognition. Part 1 (Source monitoring deficits). Schizophr Res. (2014) 152:12–9. 10.1016/j.schres.2013.06.022 23810736

[10] Harvey PO Lee J Horan WP Ochsner K Green MF. Do patients with schizophrenia benefit from a self-referential memory bias? Schizophr Res. (2011) 127:171–7. 10.1016/j.schres.2010.11.011 21147520 PMC3050992

[11] Michael J Park S. Anomalous bodily experiences and perceived social isolation in schizophrenia: an extension of the Social Deafferentation Hypothesis. Schizophr Res. (2016) 176:392–7. 10.1016/j.schres.2016.06.013 27344986 PMC8070733

[12] Nelson B Whitford TJ Lavoie S Sass LA. What are the neurocognitive correlates of basic self-disturbance in schizophrenia?: integrating phenomenology and neurocognition. Part 2 (Aberrant salience). Schizophr Res. (2014) 152:20–7. 10.1016/j.schres.2013.06.033 23863772

[13] Silver H Goodman C. Impairment in error monitoring predicts poor executive function in schizophrenia patients. Schizophr Res. (2007) 94:156–63. 10.1016/j.schres.2007.04.019 17561375

[14] Bodatsch M Brockhaus-Dumke A Klosterkötter J Ruhrmann S. Forecasting psychosis by event-related potentials-systematic review and specific meta-analysis. Biol Psychiatry. (2015) 77:951–8. 10.1016/j.biopsych.2014.09.025 25636178

[15] Newson JJ Thiagarajan TC. EEG frequency bands in psychiatric disorders: a review of resting state studies. Front Hum Neurosci. (2019) 12:521. 10.3389/fnhum.2018.00521 30687041 PMC6333694

[16] Kim JS Shin KS Jung WH Kim SN Kwon JS Chung CK. Power spectral aspects of the default mode network in schizophrenia: an MEG study. BMC Neurosci. (2014) 15:104. 10.1186/1471-2202-15-104 25189680 PMC4262086

[17] Araki T Niznikiewicz M Kawashima T Nestor PG Shenton ME McCarley RW. Disruption of function-structure coupling in brain regions sub-serving self monitoring in schizophrenia. Schizophr Res. (2013) 146:336–43. 10.1016/j.schres.2013.02.028 23507356 PMC3634126

[18] Horan WP Foti D Hajcak G Wynn JK Green MF. Impaired neural response to internal but not external feedback in schizophrenia. Psychol Med. (2012) 42:1637–47. 10.1017/S0033291711002819 22152069 PMC3559180

[19] Morris SE Heerey EA Gold JM Holroyd CB. Learning-related changes in brain activity following errors and performance feedback in schizophrenia. Schizophr Res. (2008) 99:274–85. 10.1016/j.schres.2007.08.027 17889510 PMC2329821

[20] Morris SE Yee CM Nuechterlein KH. Electrophysiological analysis of error monitoring in schizophrenia. J Abnorm Psychol. (2006) 115:239–50. 10.1037/0021-843X.115.2.239 16737389

[21] Arnfred SM Hemmingsen RP Parnas J. Delayed early proprioceptive information processing in schizophrenia. Br J Psychiatry. (2006) 189:558–9. 10.1192/bjp.bp.105.017087 17139042

[22] Arnfred SMH Mørup M Thalbitzer J Jansson L Parnas J. Attenuation of beta and gamma oscillations in schizophrenia spectrum patients following hand posture perturbation. Psychiatry Res. (2011) 185:215–24. 10.1016/j.psychres.2009.10.005 20494456

[23] Arnfred SM Raballo A Morup M Parnas J. Self-disorder and brain processing of proprioception in schizophrenia spectrum patients: a re-analysis. Psychopathology. (2015) 48:60–4. 10.1159/000366081 25401765

[24] de la Asuncion J Bervoets C Morrens M Sabbe B De Bruijn ERN. EEG correlates of impaired self–other integration during joint-task performance in schizophrenia. Soc Cogn Affect Neurosci. (2015) 10:1365–72. 10.1093/scan/nsv023 25759471 PMC4590534

[25] Jia S Liu M Huang P Zhao Y Tan S Go R. Abnormal alpha rhythm during self-referential processing in schizophrenia patients. Front Psychiatry. (2019) 10:691. 10.3389/fpsyt.2019.00691 31632304 PMC6779928

[26] Metzler S Theodoridou A Aleksandrowicz A Müller M Obermann C Kawohl W. Evaluation of trait adjectives and ego pathology in schizophrenia: an N400 study. Psychiatry Res. (2014) 215:533–9. 10.1016/j.psychres.2013.12.025 24411073

[27] Northoff G Sandsten KE Nordgaard J Kjaer TW Parnas J. The self and its prolonged intrinsic neural timescale in schizophrenia. Schizophr Bull. (2021) 47:170–9. 10.1093/schbul/sbaa083 32614395 PMC7825007

[28] Silva JR Torres WM Ortiz MS. Abnormal electrophysiological activation in schizophrenics during a personal traits attribution task. Biol Res. (2008) 41:143–50. 10.4067/S0716-97602008000200003 18949131

[29] Tikka SK Nizamie SH Das AK Agarwal N Goyal N. Schneiderian first rank symptoms in schizophrenia: a developmental neuroscience evaluation. Int J Dev Neurosci. (2016) 50:39–46. 10.1016/j.ijdevneu.2016.02.001 26952695

[30] Zhao Y Zhang D Tan S Song C Cui J Fan F. Neural correlates of the abolished self-referential memory effect in schizophrenia. Psychol Med. (2014) 44:477–87. 10.1017/S0033291713001177 23721746

[31] Hernández-García M Martín-Gómez C Gómez-García M Gomez-Pilar J Núñez-Novo P Arjona-Valladares A. Abnormal self-experiences related to a hypersynchronic brain state in schizophrenia. Schizophr Res. (2020) 222:538–40. 10.1016/j.schres.2020.03.056 32507377

[32] Nelson B Lavoie S Gaweda Ł Li E Sass LA Koren D. The neurophenomenology of early psychosis: an integrative empirical study. Conscious Cogn. (2020) 77:102845. 10.1016/j.concog.2019.102845 31678780

[33] Abhishek P Nizamie SH Dubey I Goyal N Tikka SK Pachori H. Lower P300 amplitudes for internally-generated events in patients with schizophrenia. Asian J Psychiatr. (2018) 35:67–71. 10.1016/j.ajp.2018.05.013 29787955

[34] Bühler T Kindler J Schneider RC Strik W Dierks T Hubl D. Disturbances of agency and ownership in schizophrenia: An auditory verbal event related potentials study. Brain Topogr. (2016) 29:716–27. 10.1007/s10548-016-0495-1 27209172

[35] Ford JM Mathalon DH Whitfield S Faustman WO Roth WT. Reduced communication between frontal and temporal lobes during talking in schizophrenia. Biol Psychiatry. (2002) 51:485–92. 10.1016/S0006-3223(01)01335-X 11922884

[36] Ford JM Roach BJ Faustman WO Mathalon DH. Synch before you speak: auditory hallucinations in schizophrenia. Am J Psychiatry. (2007) 164:458–66. 10.1176/ajp.2007.164.3.458 17329471

[37] Ford JM Roach BJ Faustman WO Mathalon DH. Out-of-synch and out-of-sorts: dysfunction of motor-sensory communication in schizophrenia. Biol Psychiatry. (2008) 63:736–43. 10.1016/j.biopsych.2007.09.013 17981264 PMC2330266

[38] Mathalon DH Jorgensen KW Roach BJ Ford JM. Error detection failures in schizophrenia: ERPs and FMRI. Int J Psychophysiol. (2009) 73:109–17. 10.1016/j.ijpsycho.2009.02.005 19414043 PMC4005823

[39] Mathalon DH Roach BJ Ferri JM Loewy RL Stuart BK Perez VB. Deficient auditory predictive coding during vocalization in the psychosis risk syndrome and in early illness schizophrenia: the final expanded sample. Psychol Med. (2019) 49:1897–904. 10.1017/S0033291718002659 30249315

[40] Perez VB Ford JM Roach BJ Loewy RL Stuart BK Vinogradov S. Auditory cortex responsiveness during talking and listening: early illness schizophrenia and patients at clinical high-risk for psychosis. Schizophr Bull. (2012) 38:1216–24. 10.1093/schbul/sbr124 21993915 PMC3494053

[41] Pinheiro AP Rezaii N Rauber A Nestor PG Spencer KM Niznikiewicz M. Emotional self-other voice processing in schizophrenia and its relationship with hallucinations: ERP evidence. Psychophysiology. (2017) 54:1252–65. 10.1111/psyp.12880 28474363

[42] Roach BJ Ford JM Biagianti B Hamilton HK Ramsay IS Fisher M. Efference copy/corollary discharge function and targeted cognitive training in patients with schizophrenia. Int J Psychophysiol. (2019) 145:91–8. 10.1016/j.ijpsycho.2018.12.015 30599145 PMC6616012

[43] Roach BJ Ford JM Loewy RL Stuart BK Mathalon DH. Theta phase synchrony is sensitive to corollary discharge abnormalities in early illness schizophrenia but not in the psychosis risk syndrome. Schizophr Bull. (2021) 47:415–23. 10.1093/schbul/sbaa110 32793958 PMC7965080

[44] Toyomaki A Hashimoto N Kako Y Murohashi H Kusumi I. Neural responses to feedback information produced by self-generated or other-generated decision-making and their impairment in schizophrenia. PLoS ONE. (2017) 12:e0183792. 10.1371/journal.pone.0183792 28837639 PMC5570365

[45] Whitford TJ Mathalon DH Shenton ME Roach BJ Bamer R Adcock RA. Electrophysiological and diffusion tensor imaging evidence of delayed corollary discharges in patients with schizophrenia. Psychol Med. (2011) 41:959–69. 10.1017/S0033291710001376 20663254 PMC3807011

[46] Whitford TJ Oestreich LKL Ford JM Roach BJ Loewy RL Stuart BK. Deficits in cortical suppression during vocalization are associated with structural abnormalities in the arcuate fasciculus in early illness schizophrenia and clinical high risk for psychosis. Schizophr Bull. (2018) 44:1312–22. 10.1093/schbul/sbx144 29194516 PMC6192501

[47] Bob P Susta M Glaslova K Boutros NN. Dissociative symptoms and interregional EEG cross-correlations in paranoid schizophrenia. Psychiatry Res. (2010) 177:37–40. 10.1016/j.psychres.2009.08.015 20381169

[48] Kindler J Hubl D Strik WK Dierks T Koenig T. Resting-state EEG in schizophrenia: auditory verbal hallucinations are related to shortening of specific microstates. Clin Neurophysiol. (2011) 122:1179–82. 10.1016/j.clinph.2010.10.042 21123110

[49] Knowles EEM David AS Reichenberg A. Processing speed deficits in schizophrenia: reexamining the evidence. Am J Psychiatry. (2010) 167:828–35. 10.1176/appi.ajp.2010.09070937 20439390

[50] Northoff G. Is the self a higher-order or fundamental function of the brain? The “basis model of self-specificity” and its encoding by the brain's spontaneous activity. Cogn Neurosci. (2016) 7:203–22. 10.1080/17588928.2015.1111868 26505808

[51] Northoff G Wainio-Theberge S Evers K. Is temporo-spatial dynamics the “common currency” of brain and mind? In Quest of “Spatiotemporal Neuroscience”. Phys Life Rev. (2020) 33:34–54. 10.1016/j.plrev.2019.05.002 31221604

[52] Northoff G Wainio-Theberge S Evers K. Spatiotemporal neuroscience – what is it and why we need it. Phys Life Rev. (2020) 33:78–87. 10.1016/j.plrev.2020.06.005 32684435

[53] Northoff G. Is schizophrenia a spatiotemporal disorder of the brain's resting state? World Psychiatry. (2015) 14:34–5. 10.1002/wps.20177 25655148 PMC4329887

[54] Mishara AL Lysaker PH Schwartz MA. Self-disturbances in schizophrenia: history, phenomenology, and relevant findings from research on metacognition. Schizophr Bull. (2014) 40:5–12. 10.1093/schbul/sbt169 24319117 PMC3885311

[55] Leonhardt BL Vohs JL Bartolomeo LA Visco A Hetrick WP Bolbecker AR. Relationship of metacognition and insight to neural synchronization and cognitive function in early phase psychosis. Clin EEG Neurosci. (2020) 51:259–66. 10.1177/1550059419857971 31241355

[56] Bai Y Nakao T Xu J Qin P Chaves P Heinzel A. Resting state glutamate predicts elevated pre-stimulus alpha during self-relatedness – a combined EEG-MRS study on “rest-self overlap”. Soc Neurosci. (2016) 11:249–63. 10.1080/17470919.2015.1072582 26207415

[57] Qin P Grimm S Duncan NW Fan Y Huang Z Lane T. Spontaneous activity in default-mode network predicts ascription of self-relatedness to stimuli. Soc Cogn Affect Neurosci. (2016) 11:693–702. 10.1093/scan/nsw008 26796968 PMC4814798

[58] Meyer ML Lieberman MD. Why people are always thinking about themselves: medial prefrontal cortex activity during rest primes self-referential processing. J Cogn Neurosci. (2018) 30:714–21. 10.1162/jocn_a_01232 29308983

[59] Davey CG Pujol J Harrison BJ. Mapping the self in the brain's default mode network. Neuroimage. (2016) 132:390–7. 10.1016/j.neuroimage.2016.02.022 26892855

[60] Wolff A Di Giovanni DA Gómez-Pilar J Nakao T Huang Z Longtin A. The temporal signature of self: temporal measures of resting-state EEG predict self-consciousness. Hum Brain Mapp. (2019) 40:789–803. 10.1002/hbm.24412 30288845 PMC6865612

[61] Huang Z Obara N Davis HH IV Pokorny J Northoff G. The temporal structure of resting-state brain activity in the medial prefrontal cortex predicts self-consciousness. Neuropsychologia. (2016) 82:161–70. 10.1016/j.neuropsychologia.2016.01.025 26805557

[62] Kolvoort IR Wainio-Theberge S Wolff A Northoff G. Temporal integration as “common currency” of brain and self-scale-free activity in resting-state EEG correlates with temporal delay effects on self-relatedness. Hum Brain Mapp. (2020) 41:4355–74. 10.1002/hbm.25129 32697351 PMC7502844

[63] Northoff G Gomez-Pilar J. Overcoming rest–task divide—abnormal temporospatial dynamics and its cognition in schizophrenia. Schizophr Bull. (2020):sbaa178. Advance online publication. 10.1093/schbul/sbaa178 33305324 PMC8661394

