# Supplementary material for: Abnormal ERPs and Brain Dynamics Mediate Basic Self Disturbance in Schizophrenia: A Review of EEG and MEG Studies
Source: Front Psychiatry. 2021 Apr 12;12:642469. doi: 10.3389/fpsyt.2021.642469 (PMC8072007; doi:10.3389/fpsyt.2021.642469)
Supplement: Supplementary file 1 [file Table_1.DOCX]

# Table 1: Participants in the Studies

| **Article** | **Type and Number of Patients** | **Medication** | **Healthy Controls (HCs)** | **Psychopathology Scales** |
| --- | --- | --- | --- | --- |
| Abhishek et al. (2018) | Mean duration of illness is 6.59 years; 15 with paranoid SCZ and 2 with undifferentiated SCZ (all right-handed males, various exclusion criteria) | 3 using typical antipsychotics; 14 using atypical antipsychotics | 14 right-handed males | Psychotic Symptom Rating Scale (PSYRATS); Scale for the Assessment of Positive Symptoms (SAPS); Scale for the Assessment of Negative Symptoms (SANS) |
| Araki et al. (2013) | 18 with chronic SCZ; various exclusion criteria | Average dosage: 414.2 mg chlorpromazine equivalent | 18 right-handed males | Structured Clinical Interview for DSM-IV; Positive and Negative Syndrome Scale (PANSS) |
| Arnfred et al. (2006) | 12 SCZ (all right-handed males); mean duration of illness: 9.5 years | 2 drug-naïve, the rest had used antipsychotic medication for cumulative periods ranging from 1-28 months; all had been without medication for at least the preceding month | 24 right-handed males; no significant differences in cognitive functioning compared to SCZ group | Schedules for Clinical Assessment in Neuropsychiatry (SCAN), version 2.1; SAPS; SANS |
| Arnfred et al. (2011) | Patients admitted for the first time to a specialist unit for schizophrenia spectrum disorders; 8 SCZ (of whom 6 were female, and of whom 7 were paranoid type and 1 was disorganized type) and 10 with schizotypal personality disorder (of whom 5 were female); 3 out of the total were left-handed | All medicated except 3 of the SCZ and 1 of the group with schizotypal personality disorder; SCZ mainly taking atypical antipsychotics (some typical antipsychotics and some SSRIs), while schizotypal personality group mainly taking SSRIs (some antipsychotics) | 18, matched to the patient groups by age, gender, and handedness; various exclusion criteria | DSM-IV criteria; Operational Criteria Checklist; PANSS; SAPS; SANS |
| Arnfred et al. (2015) | 6 with SCZ and 6 with schizotypal personality disorder; 7 of the total were female and 1 was left-handed; mean duration of illness (for all subjects) was 2.5 years | 2 had never used antipsychotics, 9 received modern antipsychotics, and 1 used traditional antipsychotics; 8 also treated with antidepressants | None | DSM-IV criteria, then confirmed using the Operational Criteria Checklist; Examination of Anomalous Self-Experience (EASE) |
| Bob et al. (2010) | 58 with SCZ (30 males and 28 females); partial remission within 5 years of onset of SCZ; no more than 2 hospitalizations; various exclusion criteria | Typical antipsychotics (mainly low to medium doses) | None | DSM-IV criteria, then confirmed by MINI (Mini International Neuropsychiatric Interview) version 5.0.0; PANSS; Dissociative Experiences Scale (DES) |
| Bühler et al. (2016) | 28 with SCZ or schizoaffective psychosis; 14 hallucinators (that is, had hallucinated within the last 2 weeks), and 14 non-hallucinators; 14 males and 14 females | Average dosage: 537.8 mg chlorpromazine equivalent in hallucinators, 504.5 mg chlorpromazine equivalent in non-hallucinators | 28 (with various exclusion criteria); 14 male and 14 female | PANSS; Auditory Hallucination Rating Scale (AHRS); PSYRATS |
| de la Asuncion et al. (2015) | 16 with SCZ (15 inpatients and 1 outpatient); no information on duration of illness; exclusion criteria: medication had changed within 2 weeks before, history of substance abuse within 6 months before | No information | 16 (excluded those with psychiatric history) | DSM-IV-TR criteria assessed with the Structured Clinical Interview for DSM Axis-I Disorders (SCID); SANS; SAPS |
| Ford et al. (2002) | 12 with SCZ (11 males and 1 female); 6 with undifferentiated SCZ, 4 with paranoid SCZ, 1 with disorganized SCZ, and 1 with residual SCZ; various exclusion criteria including alcohol or drug abuse within last 30 days; 4 had less recent histories of alcohol or drug abuse or addiction | 9 taking atypical antipsychotics and 3 taking typical antipsychotics | 10 (9 male and 1 female; excluded those with history of Axis 1 disorders as per DSM-IV) | SCID; Brief Psychiatric Rating Scale (BPRS); SAPS |
| Ford et al. (2007) | 24 with SCZ (4 females); various exclusion criteria; 10 with undifferentiated SCZ, 9 with paranoid SCZ, 3 with schizoaffective disorder, and 2 with residual SCZ | All taking antipsychotic medication (20 atypical, 4 typical) | 25 (6 females); various exclusion criteria | SCID; SANS; SAPS; BPRS |
| Ford et al. (2008) | 23 with SCZ (21 men, 2 women); 11 with undifferentiated SCZ, 7 with paranoid SCZ, 3 with schizoaffective disorder, 2 with residual SCZ; various exclusion criteria | Mean antipsychotic dose: 682 mg (chlorpromazine equivalent); 21 taking atypical antipsychotic medication, 2 taking typical antipsychotic medication | 25 (18 men, 7 women); various exclusion criteria | SCID; BPRS; SANS; SAPS |
| Hernández-García et al. (2020) | 25 SCZ, of which 17 were chronic and 8 were first-episode; mixture of males and females; various exclusion criteria | Stable dose of atypical antipsychotic medication (unspecified number of chlorpromazine equivalents) | None | DSV-5; PANSS; Inventory of Psychotic-Like Anomalous Self-Experiences (IPASE) |
| Horan et al. (2012) | 35 outpatients with SCZ (16 completed Study 1 and all completed Study 2); various exclusion criteria including alcohol or substance abuse or dependence within last 6 month; earlier many had had alcohol or substance abuse or dependence | 30 receiving atypical antipsychotic medication, 3 receiving typical antipsychotic medication, and 2 receiving both | 33 (14 completed Study 1 and all completed Study 2) | SCID; BPRS |
| Jia et al. (2019) | 18 with SCZ; mix of males and females, all right-handed; average duration of illness was 121 months; 7 with paranoid SCZ, 8 with undifferentiated SCZ [Note: for reasons unknown, the numbers do not appear to add up] | Medication for at least one month prior; average dosage: 498.6 mg chlorpromazine equivalent | 18 (with various exclusion criteria) | DSM-IV; PANSS |
| Kim et al. (2014) | 20 SCZ (4 female); condition stable over past year; several exclusion criteria; mean duration of illness: 5.83 years | Atypical antipsychotics | 20 (6 female); similar age and IQ to SCZ participants; none with current or past Axis I diagnosis, or with first- to third-degree relatives with psychiatric conditions, or meeting the exclusion criteria for the SCZ participants | SCID-IV; PANSS; Global Assessment of Function (GAF) |
| Kindler et al. (2011) | 8 with paranoid SCZ who experienced auditory verbal hallucinations and 1 with acute polymorphic psychotic disorder; no information on duration of illness; 3 female and 6 male; all subjects were right-handed | 2 subjects using typical antipsychotic medication, 6 subjects using atypical antipsychotic medication; 4 also received benzodiazepine or benzodiazepine-like medication [Note: for reasons unknown, the numbers do not appear to add up] | None | Clinical Global Impression Scale (CGI); PANSS; Oulis Auditory Hallucinations Rating Scale |
| Mathalon et al. (2009) | 11 with SCZ (8 male, 3 female); 9 right-handed, 1 left-handed, 1 ambidextrous; 6 with undifferentiated SCZ, 4 with paranoid SCZ, 1 with residual SCZ; various exclusion criteria | 10 taking atypical antipsychotics, 1 taking a typical antipsychotic | 10 (7 male, 3 female); all right-handed; various exclusion criteria | SCID; BPRS |
| Mathalon et al. (2019) | 71 individuals with clinical high risk (CHR) for psychosis (30 female, 41 male), 84 patients with early-illness schizophrenia (23 female, 61 male) (ESZ); mean duration of illness for ESZ: 1.85 years (maximum 5); various exclusion criteria | In CHR group, 13 taking atypical antipsychotics and 58 unmedicated; in ESZ group, 70 taking atypical antipsychotics, 2 taking typical antipsychotics, 3 taking both, and 9 unmedicated | 103 (42 female, 61 male); various exclusion criteria | Structured Interview for Prodromal Syndromes (SIPS) and Scale of Prodromal Symptoms (SOPS) for CHR; SCID, SANS, and SAPS for ESZ |
| Metzler et al. (2014) | 16 with SCZ (all right-handed); no information on duration of illness | All taking atypical antipsychotics; 1 also taking a typical antipsychotic and 1 also taking an antidepressant; average antipsychotic dosage: 668 mg chlorpromazine equivalent | 16 (all right-handed) | Standardized semi-structured interview based on the ADMP system; SANS; SAPS |
| Morris et al. (2006) | 16 patients (11 male and 5 female; 7 with undifferentiated SCZ, 3 with paranoid SCZ, 1 with disorganized SCZ, 2 with depressive-type schizoaffective disorder, and 3 with schizophreniform disorder); various exclusion criteria | 15 taking atypical antipsychotics, 1 taking a typical antipsychotic | 11 (7 male and 4 female); various exclusion criteria | SCID; BPRS; SANS |
| Morris et al. (2008) | 26 outpatients (all right-handed; 18 male, 8 female), 25 with SCZ and 1 with schizoaffective disorder | 21 taking atypical antipsychotics, 3 taking typical antipsychotics, and 2 taking both | 27 (26 right-handed, 1 left-handed; 16 male, 11 female); various exclusion criteria | SCID; BPRS; SANS |
| Nelson et al. (2020) | 50 ultra-high risk for psychosis patients and 39 first-episode psychosis patients; three groups of ultra-high-risk for psychosis patients were included: those with attenuated psychotic symptoms (37), those with brief limited intermittent psychotic symptoms (2), and those with trait vulnerability combined with deteriorating or chronic low functioning (11); first-episode patients were required to have had daily positive symptoms for at least one week but to have stabilized from acute symptoms; both groups 15-25 years old with IQ of 70 or higher, including a mixture of females and males | 15 of the first-episode psychosis group taking antipsychotic medication; 11 of the ultra-high risk group taking antidepressant medication | 34 (mixture of females and males); 15-25 years old with no psychiatric history and IQ of 70 or higher | EASE; MINI; SCID-I; SCID-II; Comprehensive Assessment of At Risk Mental States (CAARMS); BPRS; SANS |
| Northoff et al. (2020) | 34 with SCZ (mixture of males and females); diagnosis with SCZ within the last year; majority were in a stable post-acute symptomatic phase of illness; exclusion criteria: active drug abuse or any conditions likely to affect the somatosensory system or cognition | Majority were taking atypical antipsychotics; 30 taking antipsychotics, 2 taking antiepileptics; 5 taking antidepressants | 35 (mixture of males and females); same exclusion criteria as SCZ patients | PANSS; Operational Criteria Checklist for Psychotic Illness and Affective Illness (OPCRIT); perceptual domain of the Bonn-scale (BSABS); EASE |
| Perez et al. (2012) | 40 individuals at clinical high risk (CHR) for psychosis, 81 with SCZ (of which 41 had early-illness SCZ); various exclusion criteria | No information | 89; various exclusion criteria | SCID and PANSS for SCZ; SIPS and SOPS for CHR |
| Pinheiro et al. (2017) | 15 chronic SCZ patients with average duration of illness of 18.56 months; 4 with paranoid SCZ, 3 with schizoaffective disorder, 8 with unspecified form of SCZ | Average dosage: 396.91 mg chlorpromazine equivalent | 16 with no history of DSM-IV Axis 1 or Axis 2 disorders and with no first- or second-degree relatives with Axis 1 disorders; matched to patients by sex but male/female distribution not stated | PANSS; SANS; SAPS |
| Roach et al. (2019) | 49 early in the course of SCZ (maximum duration of illness = 5 years), schizophreniform disorder, or schizoaffective disorder, divided into two groups: targeted auditory training (n = 23; 7 females, 16 males) and computer games (n = 26; 6 females, 20 males) | 42 taking atypical antipsychotics, 1 taking a typical antipsychotic, 1 taking both, and 5 unmedicated; average dose (mg, chlorpromazine equivalents): 277.4 in targeted auditory training group, 422.8 in computer games group | 29 (of whom 14 were female and 28 right-handed) | SCID; SANS; SAPS |
| Roach et al. (2020) | 71 individuals meeting Psychosis Risk Syndrome (PRS), 84 individuals with early-illness schizophrenia (ESZ) | ESZ: 70 taking atypical antipsychotics, 2 taking typical antipsychotics, 3 taking both, and 9 unmedicated; PRS: 13 taking atypical antipsychotics, 58 unmedicated | 103 | For PRS, SIPS and SOPS; for ESZ, SCID, SANS, and SAPS |
| Silva et al. (2008) | 8 paranoid SCZ (all right-handed males); average duration of illness: 10.4 years; exclusion criteria: organic brain disorders, intellectual disability, substance dependence, having electroconvulsive therapy within last year | Clozapine (dose not specified) | 7 with no personal or family history of any psychiatric disorder | ICD-10 criteria |
| Tikka et al. (2016) | Two groups of SCZ patients: 20 with Schneiderian first-rank symptoms and 20 without; various exclusion criteria; all male and right-handed | Stable dose of antipsychotic medication (no further details) | 20 (all right-handed) | PANSS; first-rank symptoms were measured based on Mellor (1970), adapted to have wide and narrow definitions by O’Grady (1990) and using a specifically designed score sheet found in an appendix |
| Toyomaki et al. (2017) | 11 with SCZ (6 women and 5 men); mean duration of illness was 7.3 years | All taking atypical antipsychotics (details available on how many subjects taking which medications and what doses) | 11 (6 women and 5 men) | Structured clinical interview for Axis I disorders, described in the DSM-IV; PANSS |
| Whitford et al. (2011) | 21 with chronic SCZ (19 male, 2 female) | 17 taking atypical antipsychotics, 3 taking typical antipsychotics, and one taking both; average dose (chlorpromazine equivalents): 537.5 mg | 25 (19 male, 6 female) | BPRS |
| Whitford et al. (2018) | 51 with early-illness SCZ, defined as within 2.5 years of their first hospitalization; 40 at clinical high risk for psychosis; various exclusion criteria; mixture of males and females | Early-illness SCZ patients: 2 unmedicated, 48 taking atypical antipsychotics, 1 taking a typical antipsychotic, 1 taking both atypical and typical antipsychotics; people with clinical high risk for psychosis: 33 unmedicated, 7 taking atypical antipsychotics [Note: the numbers do not appear to quite add up, for reasons unknown] | 59 (mixture of males and females) | Early illness SCZ patients: SCID, SANS, SAPS; Clinical high-risk of psychosis: Structure Interview of Psychosis-Risk Syndromes (SIPS), including Scale of Prodromal Symptoms (SOPS), and met at least 1 of 3 sub-syndromes of the Criteria of Psychosis-Risk Syndromes (COPS) |
| Zhao et al. (2014) | 20 inpatients with SCZ (schizoaffective disorder, schizotypal and schizoid personality disorder excluded); 8 with paranoid SCZ and 12 with undifferentiated SCZ; various exclusion criteria; average duration of illness: 138.4 months; mixture of males and females | Stable medication for at least 1 month before; 18 taking atypical antipsychotics, 1 taking a typical antipsychotic, 1 taking both; average dosage: 511.2 mg chlorpromazine equivalent | 22 (mixture of males and females); various exclusion criteria | DSM-IV criteria for SCZ; PANSS |

# Table 2: Information on Study Design

| **Article** | **Concept of Self** | **Operationalization (Task)** | **Resting State Data** | **Number of EEG Electrodes / MEG Channels** | **Other Non-Behavioral Measures** |
| --- | --- | --- | --- | --- | --- |
| Abhishek et al. (2018) | Ability to differentiate internally-generated and externally-generated stimuli; they connect this to corollary discharge in the Discussion section | Source monitoring task based on Woodward et al. (2003) and Woodward et al. (2007); participants shown 60 emotionally neutral words with the letters jumbled; for 30 words a computer voice solves the word, whereas for 30 the participant must do so; immediately after, they are shown the words and must identify which they solved and which the computer solved | None | 40 EEG electrodes | None |
| Araki et al. (2013) | Self-monitoring, seen through the biological markers of error-related negativity (ERN; a component of ERPs) and correct response negativity (CRN) | Color Stroop task; participants shown stimuli in four colors and asked to press the according one of four buttons; sometimes the word matched the color, sometimes not, and sometimes the word is neutral; warning sound sometimes played over headphones during the task to increase the error rate | None | 64 EEG electrodes | Magnetic resonance imaging (MRI) |
| Arnfred et al. (2006) | Self-awareness, measured through proprioceptive ability | Changing the amount of weight on participants’ hands while wrists remain supported (maximum weight of 400g); passive paradigm with paired stimuli and active paradigm in which participants reported changes in weight | None | Not stated (recorded evoked potentials at 7 electrodes) | None |
| Arnfred et al. (2011) | Self-awareness, measured through proprioceptive ability | Changing the amount of weight on participants’ hands while wrists remain supported (maximum weight of 400g); two conditions: regularly alternating and random order | None | 64 EEG electrodes | Electromyography (EMG) |
| Arnfred et al. (2015) | Self-awareness from the first-person perspective, i.e. basic self-identity, measured through proprioceptive information processing | Proprioceptive stimuli; described further in Arnfred et al. (2011): succession of stimuli administered to the hands while the participant attempts to focus on a fixation cross on a screen in front of them | None | 64 EEG electrodes | None |
| Bob et al. (2010) | Neural binding and integration, which they postulate leads to dissociative symptoms when disturbed | None | 5 minutes of resting-state EEG with closed eyes, sitting in a chair in a quiet room | 32 EEG electrodes | None |
| Bühler et al. (2016) | Ability to distinguish between self-generated and externally generated stimuli (i.e., self-monitoring); two facets: sense of agency and sense of ownership | Six variations of a task modifying visual and/or auditory components; a) see a word, read it aloud, hear it in their own voice; b) see a word, read it aloud, and hear it in an unfamiliar voice; c) hear own voice recorded from part (a), but without having seen or spoken the word; d) hear an unfamiliar voice say a word without seeing or saying the word; e) see a word, read it aloud, and hear it after a 200 ms delay; f) see a word, read it aloud, and receive no auditory feedback | 4 minutes of resting-state EEG preceded the experiment | 74 EEG electrodes | None |
| de la Asuncion et al. (2015) | Self-other integration, a low-level ability used in joint task performance | “Social Simon task,” a variant on the classic Simon task; two participants observe stimuli and each press a button when “their” color (either red or green) appears on a screen; their reaction times are observed, and vary based on distance from a white fixation cross | None | 27 EEG electrodes | None |
| Ford et al. (2002) | Self-monitoring, as seen in corollary discharge | Talk-listen paradigm, alternating for 7 different statements, listening for 30 seconds each and speaking for 30 seconds each (total: about 7 minutes); in listening condition, heard the statement repeatedly; in talking condition, repeated the statement at a normal volume | None | 14 EEG electrodes | None |
| Ford et al. (2007) | Self-monitoring, as seen in corollary discharge | Participants said “ah” when a cue appeared on a screen, then waited for the next cue, in blocks of five cues; they then watched the screen during a rest block; after several alternating task and rest blocks, switched to listen rather than task blocks | None | 27 EEG electrodes | None |
| Ford et al. (2008) | Self-monitoring, as seen in corollary discharge | Pressing a button at will every 1-2 seconds for 2 minutes (serving as a control task for Ford et al., 2007 – see previous entry) | None | 42 EEG electrodes | None |
| Hernández-García et al. (2020) | Not specified beyond the use of IPASE, which they note correlates strongly with EASE | Three-tone auditory oddball task: target (20%), distractor (20%), and standard (60%); 600 tones randomly presented, and participants sat with their eyes closed and pressed a button each time after the target tone was played | None | 32 EEG electrodes | None |
| Horan et al. (2012) | Self-monitoring (error monitoring) | Two tasks for different ERP components; flanker task: participants press a mouse button to indicate if a middle arrow is facing left or right, with other arrows pointing in random directions on both sides; gambling task: select one of two doors, and depending if guess correctly either gain 80 cents or lose 40 cents | None | 64 EEG electrodes | None |
| Jia et al. (2019) | Self-referential processing as measured by self-referential memory | Self-referential memory task using positive and negative adjectives drawn from Yang and Wang’s Personality Trait Adjective List, with two stages; in the first stage, participants judge whether adjectives apply to them, then to other individuals, and then whether the adjectives are in bold; in the second stage, after several minutes, participants judge whether certain adjectives are among those that appeared in the first stage | None | 64 EEG electrodes | None |
| Kim et al. (2014) | Self-monitoring deficit, leading to seeing endogenous thoughts and speech as alien | Participants searched images on a screen for “Wally” from the book *Where’s Wally?* and pressed a button when they found him; simultaneously, standard tones of 50 ms were played, as well as deviant tones of 100 ms; the task lasted 6 minutes | 2.5 minutes while seated with eyes open observing a fixation cross on a screen | 306-channel MEG | MRI |
| Kindler et al. (2011) | Self monitoring, a deficit in which leads to misattribution of self-generated speech to external sources and thereby cause auditory verbal hallucinations | None, except pressing a button when auditory hallucinations began and stopped | 8 minutes while listening to their auditory verbal hallucinations | 74 EEG electrodes | None |
| Mathalon et al. (2009) | Distinguishing between self-generated and externally-generated stimuli (i.e., source monitoring) | Stimuli appeared at irregular intervals; “X” was more common and “K” was less common, and participants were told to respond only to “X” stimuli | Only for fMRI and not included in analysis | Data reported from 2 electrodes (Fz and Cz) | Functional magnetic resonance imaging (fMRI) |
| Mathalon et al. (2019) | Distinguishing between self-generate and externally-generated speech (i.e., source monitoring), which is hypothesized to be possible to corollary discharge/efference copy | Talk-listen paradigm; participants said “ah” as they chose every 1-2 seconds for the talk condition while listening to what they said through headphones in real-time; participants heard a recording of themselves from the talk condition for the listen condition | None | 64 EEG electrodes | None |
| Metzler et al. (2014) | Altered self-concept, manifested by reduced difference in reaction between trait adjectives that match their self-concept and trait adjectives that do not match their self-concept | Participants shown series of trait adjectives (as part of three-word sentences) and asked to evaluate whether they apply to them, and then later whether they apply to a close friend or relative (romantic connections were excluded); goal was comparing response times | None | 32 EEG electrodes | None |
| Morris et al. (2006) | Self-monitoring (error monitoring) | Flanker task: participants were first shown a fixation cross, followed by a triangle on either side, followed by the target triangle in the middle, and were instructed to respond with the hand corresponding to the direction of the target stimulus | None | 128 EEG electrodes | None |
| Morris et al. (2008) | Self-monitoring (error monitoring) | Learning task in which participants learned stimulus-response pairings and earned or lost 2 cents depending on whether they were correct (out of two options); three conditions: 1) stimulus-response pairs reliably associated (100% of time), 2) pairs associated only 80% of time, and 3) pairs associated randomly (50% of time) | None | 32 EEG electrodes | None |
| Nelson et al. (2020) | Minimal self disturbance, with two components: sense of ownership and sense of agency; more concretely, minimal self disturbance manifesting as source monitoring deficits and aberrant salience | Three tasks to test source monitoring deficits, two to test aberrant salience, and two for EEG measurement of source monitoring deficits and aberrant salience; for source monitoring deficits, 1) action memory task: performing or imagining performing a series of actions and attempting to remember which were performed vs. imagined, 2) word recognition test: responding to words with related words and then attempting to remember which were given vs. chosen, 3) temporal binding task: estimating the position of a clock hand when one presses a button, with estimates varying depending whether an audible tone plays and the difference between those two conditions being the final data; for aberrant salience, 1) salience attribution test: comparing reaction times to task-relevant and task-irrelevant cues; 2) babble task: repeating any real words and phrases that appear in a string of babble; source monitoring deficits in EEG used auditory button-press task: pressing a button to hear a sound (minus a motor-only condition) vs. hearing a sound without pressing a button, to index source monitoring (N1 suppression); aberrant salience in EEG used auditory oddball paradigm and measured mismatched negativity (MMN) | None | 64 EEG electrodes | None |
| Northoff et al. (2020) | Basic self (a structural instability of the first-person perspective) [this is the same as ipseity] | Two tasks; first is enfacement illusion, in which the participant performs facial recognition tasks with faces morphing into other faces on the screen (some of the faces were the participant’s own, so self-referential); second task uses the oddball paradigm, in which the participant listens to a series of tones and attempts to identify those with an aberrant length (non-self-referential) | 5 minutes of resting-state EEG while lying down, with gaze fixed at a point on the wall | 20 EEG electrodes | None |
| Perez et al. (2012) | Source monitoring speech, which is hypothesized to be carried out at the neural level by corollary discharge | Talk-listen paradigm: participants said “ah” at will every 1-2 seconds for three minutes and what they said was played to them through headphones; in the listen condition, the recording of the talk condition was played back to them but they did not speak | None | 64 EEG electrodes | None |
| Pinheiro et al. (2017) | Monitoring of self-generated speech (they hypothesize that when this monitoring is impaired, self-generated speech is mistaken for externally-generated speech, causing auditory verbal hallucinations) | Participants were played audio of 420 adjectives, 210 pre-recorded in their own voice and 210 in another voice, and each set divided into 70 positive adjectives, 70 negative adjectives, and 70 neutral adjectives; participants pressed one of three buttons after each adjective, indicating “Self,” “Other,” or “Unsure” | None | 64 EEG electrodes | None |
| Roach et al. (2019) | Ability to distinguish internally- and externally-generated sounds, as is putatively carried out through corollary discharge | Talk-listen paradigm; in talk condition, participants said “ah” voluntarily every 1-2 seconds for 187 seconds total, during which time they heard their own voice through headphones in real time; in the listen condition, they heard the recording of themselves from the talk condition, while remaining silent | None | 64 EEG electrodes | None |
| Roach et al. (2020) | Differentiating internally vs. externally generated sensations; mechanism is thought to be corollary discharge | Talk-listen paradigm; in talk condition, participants said “ah” voluntarily every 1-2 seconds for 187 seconds total, during which time they heard their own voice through headphones in real time; in the listen condition, they heard the recording of themselves from the talk condition, while remaining silent | None | 64 EEG electrodes | None |
| Silva et al. (2008) | Changes in personal identity leading to difficulty processing information regarding the self | Subjects presented with a series of adjectives; in the first condition (self condition), asked if the adjectives described them; in the second condition (other condition), asked if the adjectives described a certain friend or acquaintance; in the third condition, they simply read the words | None | 128 EEG electrodes | None |
| Tikka et al. (2016) | Living as a single defined conscious unit; ipseity | Task designed by Stirling et al. (2001); drawing patterns of simple designs and then attempting to recognize them among a series of other drawings; in test 1, drawing them out-of-sight, and in test 2, drawing them in plain view | 10 minutes of resting-state EEG while sitting with eyes closed on a reclining chair in a room with attenuated sound and light | 256 EEG electrodes | Serum brain derived neurotrophic factor (serum BDNF) |
| Toyomaki et al. (2017) | Sense of agency with regards to external events caused by self-generated decisions | Gambling task with two stages: self-generated decisions (in which participants picked one of two buttons) and other-generated decisions (in which they were told which button to pick) | None | 5 EEG electrodes | None |
| Whitford et al. (2011) | Ability to distinguish between self-generated and externally-generated events | Each participant was recorded saying “ah”; this was used as the auditory stimulus for a button-press task, with several conditions; participants pressed a button and heard their voice immediately, after 50 ms, after 100 ms, automatically without them pressing the button, or not at all even though they pressed the button | None | 42 EEG electrodes | Diffusion tensor imaging (DTI) |
| Whitford et al. (2018) | Suppression of self-generated speech in the primary auditory cortex | Talk-Play paradigm (see Ford et al., 2010); participants repeatedly say “ah” for 187 seconds, then listened to the recording of them speaking | None | 64 EEG electrodes | DTI and diffusion tensor tractography |
| Zhao et al. (2014) | Self-referential memory effect, i.e., the ability to remember stimuli processed with reference to the self better than hose processed without reference to the self | Self-referential memory task adapted from Mu & Han (2010), with an encoding phase and a recognition phase; in the encoding phase, participants were shown trait adjectives and asked whether they described themselves, whether they described a certain well-known person, or whether they were in bold font or not; after a break of 40 minutes, in the recognition phase, they attempt to remember if adjectives were among those included in the encoding phase | None | 57 EEG electrodes | None |

# Table 3: Results of the Studies

| **Article** | **Event-related Potential (ERP) Components** | **Frequency Bands** | **Other Measures** | **Brain Regions Involved** | **Behavioral Data** | **Relationship to Psychopathology Scales** |
| --- | --- | --- | --- | --- | --- | --- |
| Abhishek et al. (2018) | Analyzed data for P300 amplitude and latency over six brain regions for both internal source-monitoring and external source-monitoring; the only statistically significant difference between SCZ patients and HCs was lower amplitude for internal source-monitoring among SCZ patients | None | None | EEG for six brain regions was analyzed: right central, mid central, left central, right parietal, mid parietal, and left parietal; lower P300 amplitude for internal source-monitoring among SCZ patients was in the left parietal and right parietal regions | None | Not measured |
| Araki et al. (2013) | SCZ patients had significantly higher CRN amplitudes than healthy controls; unlike in past studies, no significant difference in ERN amplitudes, though this may be due to small sample size | None | MRI data revealed that patients with SCZ and healthy controls did not differ significantly in gray matter volume in any region of the ACC; no significant correlation between CRN amplitude and grey matter volume, but significant negative correlation between ERN amplitude and grey matter volume | For the MRI data, divided the anterior cingulate cortex (ACC) into left and right and then further subdivided into cognitive, affective, and subgenual regions; the negative correlation between ERN amplitude and grey matter volume was of the left ACC, at electrodes F3, Fz, F4, and C4 | Error rate on the task of 10.9% in SCZ patients and 6.4% in healthy controls, but the difference was not statistically significant | Not measured |
| Arnfred et al. (2006) | No differences in active paradigm, and no difference in P50 gating or P300 amplitude; after first stimulus, greater P60 latency in SCZ than HCs and greater P200 amplitude in SCZ than HCs; after second stimulus in passive condition, greater N70 amplitude in SCZ than HCs | None | None | P60 at left parietal electrode; P200 at central and contralateral (i.e., left) electrodes; N70 at frontal electrode | None | Not measured |
| Arnfred et al. (2011) | None | Higher gamma evoked absolute amplitude (avWT) in SCZ than HCs; reduced beta avWT in SCZ in contralateral but not frontal regions; trend towards lower gamma phase-locking factor (PLF), but not significant, driven by the regular (alternating) condition; lower beta PLF without interaction effect for condition | None | As noted, beta avWT reduced in contralateral but not frontal regions; lower beta PLF was also contralateral | None | Not measured |
| Arnfred et al. (2015) | None | Gamma frequency and beta parietal and frontal amplitude (results reported only for correlations with EASE – see rightmost column) | None | Gamma measured in unspecified region, peaking at electrode CP3; beta measured separately in parietal and frontal regions, peaking at electrodes CP3 and C1 respectively | None | Higher EASE scores correlated with no or lower gamma frequency (of maximum amplitude in gamma window-of-interest) and higher parietal and frontal beta amplitude |
| Bob et al. (2010) | None | None | Neural integration was calculated using Spearman (rank) correlation coefficients between pairs of electrodes, then compared to psychopathology scales (see rightmost column) | EEG pairs spanning many regions, using 8 EEG channels (despite a 32-channel EEG cap): F3, F4, T3, T4, C3, C4, P3, P4 | None | Relationship of EEG data to DES scores, positive symptom scores from PANSS, and negative symptom scores from PANSS; 9 of 16 EEG pairs showed significant correlation with DES, whereas only 1 did with PANSS positive symptoms and 0 with PANSS negative symptoms; Mann-Whitney test show significant differences with low and high DES scores, but no difference for low and high positive symptom or negative symptom scores |
| Bühler et al. (2016) | Analysis of two components, N100 (which they define as 116-170ms) and a late component (which they define as 172-356ms); N100 amplitude was smaller in both patient groups than in HCs; spatial filtering through template-based analysis showed that significantly more of the N100 could be attributed to the agency effect in HCs than in either patient group; template-based analysis of the late component, however, showed no significant differences between HCs and either patient group, but showed significantly more of the late component could be attributed to the agency effect in SCZ with hallucinations than SCZ without them; no significant differences between groups in ownership effect for either component; TANOVAs (topographic ANOVAs) of the N100 showed a significant agency effect in both HCs and SCZ with hallucinations, but not in SCZ without hallucinations, whereas there was no significant ownership effect in any group; TANOVAs of the late component showed a significant agency effect for HCs and SCZ with hallucinations, but not for SCZ without hallucinations, whereas the ownership was found in HCs and SCZ without hallucinations, but not for SCZ with hallucinations | None | Global field power analysis showed significant interaction between participant group and experimental condition for both components, and this held true between HCs and SCZ without hallucinations for both components, as well as between HCs and SCZ with hallucinations for the late component only | In the TANOVAs, N100 was negative in the central region (associated with sense of agency) and positive in both halves of the temporal region (associated with sense of ownership), while the late component of the ERP was negative in occipitoparietal regions and positive in bilateral frontal regions | No statistically significant difference between groups in mean voice onset time | Neither PANSS nor PSYRATS showed a statistically significant correlation with ownership or agency effects during the N100 component or the late component; that said, during the late component, PANSS showed *almost* significant agency and ownership effects for SCZ with hallucinations, with a negative correlation with agency (*p* = 0.060) and an almost significant positive correlation with ownership (*p* = 0.090) |
| de la Asuncion et al. (2015) | SCZ showed decreased no-go P3 amplitudes compared to HCs; group-condition interaction: HCs showed increased P3 amplitude in joint condition compared to individual condition, while there was no significant difference between conditions for SCZ; P3 amplitude significantly reduced in joint condition for SCZ patients, but however not in individual condition | None | None | Smaller amplitudes at electrode Fz than at electrode Cz | Faster response times by healthy controls than by patients with SCZ in both individual and joint conditions; compatibility effect that indicates the Social Simon Effect was found only in the joint condition | Not measured |
| Ford et al. (2002) | None | For HCs but not SCZ, in delta and theta bands, greater frontal-temporal coherence during talking condition than listening condition; theta power was greater during talking condition than listening condition at every site in both groups | None | The frontal-temporal coherence was reported specifically for lateral frontal and posterior temporal sites | None | None |
| Ford et al. (2007) | N100 amplitude reduced in HCs in speech condition compared to listening condition, but no such reduction in SCZ | None | Results were based on three frequency bins: 15.625 Hz, 31.25 Hz, and 46.875 Hz; greater intertrial coherence before talk condition than listening condition in both groups, though greater difference in HCs; intertrial coherence before speech (-100 ms to -75 ms) strongly correlated with N100 amplitude in HCs but not SCZ (controlling for intertrial coherence in other time bins) | Correlation between hallucination severity and pre-speech intertrial coherence was strongest at frontal-central sites; N100 results not correlated with hemisphere | No important results | Inverse correlation between intertrial coherence before speech condition and hallucination severity (in a multiple regression analysis; avolition/apathy was also included but did not produce a significant result) |
| Ford et al. (2008) | Interaction effect with localization for somatosensory ERP (<50 ms) – see fourth column | HCs had greater phase-locking factor (PLF) than SCZ in the gamma band, but no significant difference in the beta band (these measures were both prestimulus) | In HCs but not SCZ, in the left hemisphere only, phase-locking factor (combined beta and gamma) was significantly greater before pressing the button (i.e., prestimulus) than after (i.e., poststimulus) | Somatosensory ERP was larger over left hemisphere than right hemisphere in SCZ, but no significant difference in HCs; gamma phase-locking factor correlated positively with ipsilateralization of the somatosensory ERP in HCs but not SCZ (no effect in beta band) | Once three subjects who pressed the button more than twice as fast as all others were removed, no significant difference between SCZ and HCs on button press speed | Prepress beta synchrony in left hemisphere was significantly negatively correlated to avolition/apathy (from SANS), whereas gamma synchrony was not significantly related to any psychopathology scale or sub-scale; antipsychotic medication dose not correlated with prepress PLF for either beta or gamma |
| Hernández-García et al. (2020) | None | None | Spectral entropy (SE) modulation (task window minus pre-stimulus window); connectivity strength (CS; modulation measured as difference between the same two windows); results reported only in relation to psychopathology scales (see final column) | Not discussed | None | Significant positive correlation between pre-stimulus CS and IPASE (as well as two of its four sub-scales: consciousness and demarcation/transitivism), but significant negative correlation between CS modulation and IPASE (as well as the demarcation/transitivism sub-scale); after principal components analysis of the SE data, first factor (representing an increase during task over the pre-stimulus window) correlated positively with IPASE (as well as two of its four sub-scales: self-awareness and somatization) |
| Horan et al. (2012) | Smaller difference between ERN and CRN in SCZ than HCs (i.e., due to lower ERN and higher CRN, though these effects may not on their own be significant); no significant difference between groups for Pe (error positivity); no significant difference in Experiment 2 across groups for difference in FN (feedback negativity) between reward and non-reward trials | None | None | Not discussed | No significant difference in accuracy, but significantly slower response times in SCZ than HCs | Higher scores on the positive symptoms scale of the BPRS correlated with greater difference between reward and non reward trials in FN amplitude |
| Jia et al. (2019) | None | In the 100-300 ms post-stimulus band, alpha oscillations had significantly more evoked power in HCs than SCZ; no significant difference was found in beta and theta ranges; self-referential memory bias correlated positively with evoked power of alpha oscillations in SCZ patients but not healthy controls; phase lag index: significantly higher functional connectivity in alpha oscillations in the 100-300 ms window in healthy controls than patients, but not in other time windows (in all three conditions using time courses, and in self-referential and other-referential conditions using brain-network functional connectivity) | In 100-300 ms band, during self-referential condition, SCZ had lower characteristic path length and higher global efficiency than HC, but no differences in other two conditions; evoked power in alpha oscillations correlates strongly with global efficiency and characteristic path length in SCZ but not HCs | Differences in alpha oscillations from 100-300 ms post-stimulus were observed in frontal and parietal regions; significantly decreased brain-network functional connectivity in the self-referential condition in patients in central parietal, temporal, and occipital regions; localized differences in nodal efficiency between SCZ and HCs, varying between the three conditions | Faster reaction times for the physical condition than the self-referential and other-referential conditions, and significant difference in reaction times between groups; no significant difference between SCZ and HCs on recognition scores for physical or other-referential conditions, but HCs scored significantly higher than SCZ for the self-referential condition; intact self-referential memory (SRM) effect for controls, i.e., better recognition in self-referential condition than other-referential condition, but no SRM effect for patients | None |
| Kim et al. (2014) | None | Regional power spectral density significantly higher at rest in SCZ than HC; in posterior cingulate cortex, increased theta, alpha, and beta rest-task difference in SCZ compared to HC, while no significant difference in gamma; in the medial prefrontal cortex, lower difference in parts of the gamma spectrum between rest and task in SCZ compared to HC, while no significant difference for other frequency bands | Overall default mode network activity (difference between rest and task, combining frequency bands) stronger in SCZ than HC; strong coherence between posterior cingulate cortex and medial prefrontal cortex, though this was significantly reduced in SCZ for gamma | In both SCZ and HC, spatial map revealed by MEG frequency bands (rest-task difference) was similar to default mode network areas reported in other studies: posterior cingulate cortex extending to the precuneus, dorsomedial prefrontal cortex, and lateral cortex; regional power spectral density differences at rest were in the ventral prefrontal cortex and the posterior cingulate cortex | None | No statistically significant difference between groups in power at rest; for rest-task difference, significant correlation between gamma power in the medial prefrontal cortex and positive syndrome scale of PANSS, but not in the posterior cingulate cortex |
| Kindler et al. (2011) | None | None | Number of periods with auditory verbal hallucinations (AVH) and number of periods without AVH (mean of 12 with AVH and 12 without AVH); using 2-tailed *t*-tests, microstate D was found to be significantly shorter during AVH then when AVH were not occurring, whereas there were no significant relationships to the other tests | Microstates A and B were diagonally oriented, microstate C had an anterior-posterior orientation, and microstate D had a fronto-central extreme location | None (no task except pressing buttons to indicating beginning and end of AVH) | Not discussed |
| Mathalon et al. (2009) | SCZ exhibited a smaller ERN amplitude than HCs | None | None | ERN amplitude was greater at Cz than Fz, with this difference more pronounced in HCs than SCZ | SCZ showed a trend toward lower accuracy than HCs, but it was not significant (p = 0.09) | Not reported |
| Mathalon et al. (2019) | HCs exhibited greater N100 suppression than early-illness schizophrenia patients (ESZ) or the clinical high risk for psychosis group (CHR); for the HC/ESZ comparison, this was shown to be due to larger N100 in the listen condition among HCs; N100 suppression increased with age for HCs, but not for ESZ | None | None | Measured at Cz | None | No significant correlations in the ESZ group; in the CHR group, (age-corrected) N100 suppression was inversely correlated with unusual thought content |
| Metzler et al. (2014) | Trend for interaction between congruence and group in 400-600 ms and 300-500 ms windows, but not significant; in the self-referential condition, HCs had a significantly more negative N400 when adjectives were incongruent (rather than congruent), but no such effect in SCZ | None | None | The effect of controls having a significantly more negative N400 for incongruent adjectives in the self-referential condition (unlike SCZ patients) was most pronounced at the central electrode (Cz) | Response times: SCZ showed no difference between self-referential and other-referential conditions whereas controls were faster in the self-referential condition; controls responded significantly faster to positive congruent adjectives than negative congruent adjectives, whereas there was no significant difference in SCZ; separate from the responses times, in the self-referential condition, SCZ were significantly more likely to agree with negative adjectives than controls | Smaller N400 effect was correlated with greater scores for ego pathology, with no correlation between medication dosage and N400 effect; no significant effects for negative factor, psychotic factor, or disorganized factor |
| Morris et al. (2006) | Smaller ERN in SCZ than HCs in accuracy condition but not speed condition; larger CRN in SCZ than HCs in the speed condition (and a trend toward larger CRN in the accuracy condition); both groups had a greater ERN following errors in the accuracy condition than following errors in the speed condition (though the effect was much larger in HCs); no difference in Pe in either condition between groups | None | None | Both ERN and Pe were maximal at FCz, and all other analyses (see column 1) were done using that site | No significant main effect for group in terms of accuracy, though (unexpectedly, the authors note) SCZ had higher accuracy in one condition (the interference condition); SCZ had significantly lower response times than HC, overall and in the accuracy condition | Not reported |
| Morris et al. (2008) | Reduced response negativity (RN) in SCZ compared to HCs in all three conditions, and reduced feedback negativity (FBN) in SCZ compared to HCs in the 100% condition in early trials within a block (as well as a trend toward significance for reduced FBN in SCZ in 80% and 50% conditions in later trials within a block) | None | None | Not included | SCZ had lower accuracy in some conditions than HCs, but the difference was not significant; SCZ group trended towards slower response times than HCs, but the difference was not significant; SCZ took more trials to reach the criterion in the 100% condition but not the 80% condition | Reality distortion symptoms (a sum of several sections of the BPRS) correlated negatively with RN amplitude; negative symptoms (a sum of thee other sections of the BPRS) correlated negatively with FBN amplitude, and likewise negative symptoms as measured by SANS correlated negatively with FBN amplitude |
| Nelson et al. (2020) | Auditory button-press task: N1 suppression (difference in N1 component amplitude between active and passive conditions) was reduced in first-episode psychosis patients compared to ultra-high risk group and HC; auditory oddball: no significant differences in MMN amplitude between groups | None | None | N1 suppression measured at electrode Cz; same pattern at all frontocentral electrodes for MMN | No differences between groups on general measures of cognitive ability; for source monitoring, 1) action memory task: both clinical groups more likely than HC to falsely remember imagined actions as having been performed (though no significant difference for the opposite), 2) word recognition task: no significant group differences, and 3) temporal binding task: significant difference between groups, with HC showing positive temporal binding effect (perceiving the time of a button press as closer to an audible tone as compared when there is no tone), but clinical groups showing negative effect; for aberrant salience, 1) salience attribution test: significant difference on only one of four measures, and only between ultra-high risk group and HC, with HC responding faster to relevant information, 2) babble task: no significant difference | Using composite scores of all cognitive and EEG measures for source monitoring and aberrant salience, EASE was shown to account for 39.8% of the variance in source monitoring (more than any psychopathological scale) but only 6% of the variance in aberrant salience; regression analysis with EASE scores on one side and cognitive results and EEG measures on the other side, for source monitoring only, with post hoc tests for the three participant groups, found many significant correlations between EASE and task results, though most were not significant after correction for multiple comparisons; for aberrant salience, comparison of tasks to psychopathology scales yielded a number of significant correlations, but again most disappeared once multiple comparisons were adjusted for |
| Northoff et al. (2020) | None | None | Auto-correlation window (ACW) was longer and power-law exponent (PLE) was higher among SCZ than among HC during the (self-referential) enfacement illusion paradigm but not during the (non-self-referential) auditory oddball paradigm; rest-task difference was similar for SCZ and HC in the auditory oddball paradigm, but for the enfacement illusion there was a significantly smaller rest-task difference for SCZ than for HC | Not discussed | None | No direct relationships between ACW or PLE and psychopathological scales for either paradigm; however, in the enfacement illusion paradigm, ACW and PLE mediated the relationship between self-disorder and negative symptoms (where ACW and PLE values were low, there was a negative correlation between self-disorder and negative symptoms, whereas when they were high, there was a positive correlation) |
| Perez et al. (2012) | Greater N1 suppression in HCs than SCZ; when the sample was broken down into three groups, there was a significant difference between HCs and the early-illness schizophrenia (ESZ) group, but not between the clinical high risk for psychosis (CHR) group and either HCs or ESZ | None | None | Not reported | None | No significant relationship between age-corrected N1 suppression and duration of illness; no significant correlation between PANSS positive or negative scales and N1 suppression in the SCZ group, or between SOPS positive or negative scales and N1 suppression in the CHR group |
| Pinheiro et al. (2017) | Analyzed N1, P2, and late positive potential (LPP) components; no significant differences between groups on N1; among HCs, P2 amplitude was greater in self condition than other condition when the valence was positive but not for neutral or negative valences, whereas among SCZ, P2 amplitude was lower in the self condition compared to the other condition, only for neutral speech (only at the frontocentral electrode); LPP amplitude was reduced for negative speech in SCZ compared to HCs (for both self and other conditions, and specifically at the centroparietal electrode sites); among HCs, LPP amplitude was higher in self condition for positive and negative valence, but not neutral valence, while the same effect was found in SCZ but only for negative valence | None | None | P2 amplitude was larger in frontocentral and central regions than in the centroparietal region | No significant group difference accuracy; the healthy controls had no significant difference in accuracy based on the valence of the adjectives (positive, neutral, negative), but the patients with SCZ were significantly worse when the adjectives were negative | LPP amplitude for negative adjectives in non-self speech condition was positively correlated with PANSS hallucination severity (p = 0.044); the magnitude of the difference between negative and positive adjectives in the other condition was positively correlated with higher severity on the SAPS voices conversing score (p = 0.035 after Holm-Bonferroni method); by contrast, P2 amplitude was not predicted by PANSS hallucination severity |
| Roach et al. (2019) | Both SCZ treatment groups had significantly reduced N100 suppression compared to HCs before and after treatment; the targeted auditory training group did shown significantly increased N100 suppression after treatment, however; strong inverse relationship between N100 suppression at baseline and N100 suppression change after treatment (i.e., if doing worse before, show greater positive change, vs. if doing better before, show greater negative change); no change in N100 suppression scores in HCs | None | None | Not discussed | Interaction effect of time by treatment on global cognition, but post hoc tests did not find treatment group to be significant | Greater improvement on N100 suppression was associated with the worsening of bizarre behavior ratings on SAPS in the targeted auditory training group, but this was driven by two outliers |
| Roach et al. (2020) | N1 suppression in HCs; main effect of group for effect size (across N1 suppression, theta ITC suppression, and theta power suppression), as well as group/measure interaction (post-hoc tests finding only HC vs ESZ, power vs ITC to be significant, with ITC suppression being significantly greater) | Theta ITC suppression in HCs (double the effect size of N1 suppression) and theta power suppression in HCs; significantly higher theta ITC suppression in HCs than ESZ; [repeated from previous column because it also includes frequency bands:] main effect of group for effect size (across N1 suppression, theta ITC suppression, and theta power suppression), as well as group/measure interaction (post-hoc tests finding only HC vs ESZ, power vs ITC to be significant, with ITC suppression being significantly greater); both theta suppression measures were correlated with N1 suppression, though with more than double the effect size for ITC suppression, and this effect being more significant in HCs than ESZ (no significant difference between HCs and high-risk group) | None | Not reported | None | Within the ESZ group, negative correlation between severity of delusions and theta ITC suppression; theta power suppression was not significantly correlated with any symptom scale; both these tests controlled for N1 suppression; no age/group interaction was found to be significant, unlike in Roach et al. (2019) |
| Silva et al. (2008) | Specific ERP components not specified, only time windows; from 180-230 ms [identified in the figures as P200], SCZ had significantly lower amplitude in self condition than HC, whereas no significant difference in other condition; no significant effects for 310-380 ms, 415-480 ms, or 75-175 ms | None | None | 180-230 ms, 310-380 ms, and 415-480 ms windows of interest were measured at frontomedial electrodes; 75-175 ms window of interest was measured at parieto-occipital window of interest | Not reported | None |
| Tikka et al. (2016) | None | Gamma spectral power was significantly higher for SCZ with first-rank symptoms than for those without and HC in three of eight regions of interests (see fourth column); age, duration of illness, and medication dose were not significant predictors, and the effects remained highly significant when these variables were controlled; in one additional region of interest, both SCZ groups had significantly higher gamma spectral power than HC | None | The eight regions of interest for the gamma spectral power analysis were (each in both hemispheres): inferior frontal gyrus, dorsolateral prefrontal cortex, inferior parietal lobule, and superior temporal gyrus; the regions with higher gamma spectral power for SCZ with first-rank symptoms were right inferior frontal gyrus, right inferior parietal lobule, and right superior temporal gyrus; the region with higher gamma spectral power was the left superior temporal gyrus | Both groups of SCZ performed worse than the HC on both the self-monitoring task and the recognition memory task; on the self-monitoring task but not the recognition memory task, the SCZ with first-rank symptoms performed worse than those without them | Among SCZ with first-rank symptoms, FRS (narrow criteria) and PANSS positive scores were negatively correlated with outcomes on the self-monitoring task; in three brain regions, gamma spectral power was correlated positively with either FRS (narrow criteria) or PANSS (positive) |
| Toyomaki et al. (2017) | Positive deflection at ~350ms post-stimulus in all conditions; mean amplitudes larger for both groups in self condition than other condition; when the decision was made by the subject (self condition), significantly lower mean amplitude among SCZ than HC for both gain and loss conditions, but when the decision was not made by the subject (other condition), no difference between groups for either gain or loss conditions; significantly larger mean amplitude for SCZ than HC when comparing difference between conditions (self condition minus other condition), for both gain and loss outcomes | In the self-decision condition, significantly larger increase in alpha and theta spectral power in HC than SCZ from 100-500 ms | None | Electrodes C3, Cz, and C4 were the site of significantly higher alpha and theta spectral power in HC than SCZ from 100-500 ms | Probability of selecting advantageous choice in gambling task was 0.73 in healthy controls and 0.64 in patients with SCZ, but the difference was not found to be statistically significant | Not discussed |
| Whitford et al. (2011) | Significantly lower N1 suppression in SCZ than HCs in no-delay condition (as reported in previous findings); in 50 ms delay condition, significantly *higher* N1 suppression in SCZ than HCs; no significant difference in 100 ms delay condition | None | None | Not reported | None | None |
| Whitford et al. (2018) | N1 suppression (reduced N1 amplitude for self-generated speech compared to externally-generated speech) was significantly lower among SCZ than HC; clinical high-risk patients were in between, established by a linear contrast analysis (p = 0.003) | None | Diffusion tensor imaging (DTI) found significantly lower fractional anisotropy and significantly higher radial diffusivity in SCZ (but not clinical high-risk patients) than HC; tested the above values for correlation with N1 suppression, finding a negative correlation with radial diffusivity | The significant differences between SCZ and HC found through DTI were situated in the arcuate fasciculus, with no significant differences in the pyramidal tract; correlation with N1 suppression was measured at electrode Cz, and the radial diffusivity measured at the arcuate fasciculus | None | None |
| Zhao et al. (2014) | Significantly higher P2 latency and lower P2 amplitude in SCZ than HC; longer N2 latency in HC than SCZ; N2 amplitude higher in self-referential condition than in other-referential or font conditions in SCZ, whereas amplitude was higher for both self-referential and other-referential conditions than the font condition in HC; no group difference in P3 amplitudes, but larger amplitude for self-referential condition than other-referential condition, and larger amplitude for font condition than either of the other two; smaller positive slow wave (~800-1200ms post-stimulus) amplitude for SCZ than HC; in SCZ, self-referential memory effect correlates negatively with P3 amplitude in the self-referential condition in the parietal cortex (especially the right parietal cortex), while no ERP components were significantly correlated with it in HC | None | None | As noted, parietal cortex P3 amplitude (especially in right hemisphere) correlates negatively with self-referential memory effect in SCZ, but not in HC | In the encoding phase, SCZ were significantly slower than HC (and both groups were significantly faster in the font condition than the other two); in the recognition phase, SCZ performed significantly lower than HC in the self-referential condition but not in the other-referential condition, and did not show a significant self-referential memory effect, whereas HC did | Patients with paranoid SCZ performed significantly worse than those with undifferentiated SCZ in both the self-referential and other-referential conditions; P2 amplitude was lower in those with undifferentiated SCZ than in HC, but there was no significant difference between paranoid SCZ and HC; N2 latency was longer in HC than in those with paranoid SCZ, but not than those with undifferentiated SCZ; age and duration of illness were each found to be significantly correlated with results at one electrode each; no significant correlations with medication dose |
